# Temporal and spatial changes in benthic invertebrate trophic networks along a taxonomic richness gradient

**DOI:** 10.1002/ece3.8975

**Published:** 2022-06-05

**Authors:** Julie A. Garrison, Marie C. Nordström, Jan Albertsson, Francisco J. A. Nascimento

**Affiliations:** ^1^ 405054 Department of Ecology, Environment and Plant Sciences Stockholm University Stockholm Sweden; ^2^ 1040 Environmental and Marine Biology Åbo Akademi University Åbo Finland; ^3^ Umeå Marine Sciences Centre Umeå University Hörnefors Sweden; ^4^ 405054 Baltic Sea Centre Stockholm University Stockholm Sweden

**Keywords:** Baltic Sea, benthic ecology, food webs, long‐term monitoring, macrofauna

## Abstract

Species interactions underlie most ecosystem functions and are important for understanding ecosystem changes. Representing one type of species interaction, trophic networks were constructed from biodiversity monitoring data and known trophic links to assess how ecosystems have changed over time. The Baltic Sea is subject to many anthropogenic pressures, and low species diversity makes it an ideal candidate for determining how pressures change food webs. In this study, we used benthic monitoring data for 20 years (1980–1989 and 2010–2019) from the Swedish coast of the Baltic Sea and Skagerrak to investigate changes in benthic invertebrate trophic interactions. We constructed food webs and calculated fundamental food web metrics evaluating network horizontal and vertical diversity, as well as stability that were compared over space and time. Our results show that the west coast of Sweden (Skagerrak) suffered a reduction in benthic invertebrate biodiversity by 32% between the 1980s and 2010s, and that the number of links, generality of predators, and vulnerability of prey have been significantly reduced. The other basins (Bothnian Sea, Baltic Proper, and Bornholm Basin) do not show any significant changes in species richness or consistent significant trends in any food web metrics investigated, demonstrating resilience at a lower species diversity. The decreased complexity of the Skagerrak food webs indicates vulnerability to further perturbations and pressures should be limited as much as possible to ensure continued ecosystem functions.

## Graphical abstract

We utilize Swedish national yearly monitoring of benthic invertebrates to construct trophic networks from known feeding links in four basins of the Baltic Sea (the Bothnian Sea, Baltic Proper, Bornholm Basin, and Skagerrak) at 26 stations covering the whole salinity gradient of the Baltic Sea (from 4 to 34 PSU). We modeled 12 trophic network metrics (species richness, number of links, linkage density, connectance, generality, vulnerability, mean path length, shortest path, trophic level, omnivory, number of motifs, and modularity), for the intervals of time 1980–1989 and 2010–2019. We found that the Skagerrak basin, with the highest salinity, showed 32% reduction in biodiversity during the study period, which significantly decreased the number of links, linkage diversity, generality, vulnerability, and number of motifs, indicating increasing instability for the food webs and subsequent ecosystem functioning.

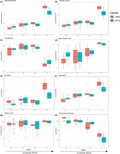

## 1 INTRODUCTION

Trophic networks, or feeding interactions within an ecosystem arranged into a food web, are a useful tool to understand population dynamics of multiple species and community organization across spatial and temporal scales (Roslin & Majaneva, 2016). The study of how food webs are structured allows better assessment of ecosystem stability and resilience to internal and external pressures (Saint‐Béat et al., 2015). Quantifying changes in food web topology using metrics, such as measures of species richness, connectance, vulnerability, and generality, have increased the knowledge of how different pressures affect different parts of the food web disproportionately (Gibert, 2019; Nordström & Bonsdorff, 2017). Anthropogenic pressures result in changes in species richness and composition through not only environmental filtering but also through biological interactions. For example, eutrophication not only directly influences the primary producers but also indirectly other trophic levels through bottom‐up trophic cascades that often result in simplified communities (Jochum et al., 2012; Nordström & Bonsdorff, 2017; Shurin et al., 2012), which can be visualized through increased trophic network connectance, or proportion of possible links realized. On the other hand, overfishing reduces top predators, resulting in a top‐down trophic cascade (Gilarranz et al., 2016; Myers & Worm, 2003; Pace et al., 1999; Pauly, 1998), which can increase middle trophic level biomass through predation pressure release, and overgraze primary producers (Elmgren et al., 2015; Pace et al., 1999). Such trophic network changes can be visualized through decreased vulnerability, or mean number of predators per prey, and shorter trophic chains. Climate warming has been correlated with increased connectance and increased variability in generality, or mean number of prey species per predator (Kortsch et al., 2019). In addition, pressures can act disproportionately on taxa, such as habitat destruction impacting sessile and long‐lived species more than mobile short‐lived ones (Bradshaw et al., 2012). Thus, when external pressures act on one organism, indirect changes to the trophic network can be predicted through trophic cascades (Shurin & Seabloom, 2005). For these reasons, trophic networks are ideal candidates for monitoring and management (Gray et al., 2014).

However, there are challenges to integrating trophic networks into management applications. Long‐term data observations with high taxonomic resolution at multiple trophic levels are commonly lacking over larger spatial scales to characterize trends in trophic networks. In addition, food webs are dynamic, and links vary over spatial and temporal scales, which often results in a necessary trade‐off between resolution (i.e., all species and links are known) and scale (McMeans et al., 2015; Thompson et al., 2012). Importantly, food web structure is not always easily inferred based on species richness or community composition alone (Frelat et al., 2022), advocating for detailed assessments of trophic interaction structure to complement those of taxonomic community structure that is commonly the focus in monitoring.

Benthic invertebrate species composition and trophic interactions vary with environmental conditions and provide a range of ecosystem functions (Johannesson & André, 2006; van der Putten et al., 2004; Winder & Schindler, 2004). Detritus dominates the diet of many benthic invertebrates, and this processing of detritus contributes to nutrient recycling and mineralization of organic matter in interactions with microbes (Dossena et al., 2012; Lauringson et al., 2014; Moore et al., 2004). Detritivores dominate soft sediments, one of the largest habitats in the world, covering 80% of the ocean floor, but are one of the least studied ecosystems in terms of trophic networks (Lenihan & Micheli, 2001; Nordström & Bonsdorff, 2017; Nybakken & Bertness, 2005). For these important ecosystem components, much remains unclear about how large‐scale or long‐term changes in benthic communities are manifested in ecological interaction structure and what the potential consequences are for functioning at the base of the food web.

The Baltic Sea has strong salinity, temperature, and species richness gradients over a relatively small geographic distance (Leppäranta & Myrberg, 2009; Snoeijs‐Leijonmalm et al., 2017), and anthropogenic pressures vary as well, both by spatial and temporal scales. The effects of the interactions between these environmental gradients and pressures can be well studied through existing research, monitoring, and governmental structures in the Baltic Sea (Reusch et al., 2018). The physical and biological gradients found in the Baltic (e.g., salinity, temperature, and species richness) allow for a better understanding of impacts of various anthropogenic pressures such as eutrophication and climate change on aquatic ecosystems (Reusch et al., 2018). Pressures like climate change are particularly relevant in the Baltic Sea, where sea surface temperatures are rising three times faster than the global mean sea temperature increase (>1°C per decade; Belkin, 2009; Reusch et al., 2018). As a result, we can expect direct and indirect impacts to food webs, shown through smaller organism body size, behavioral changes such as shifted habitat preference (poleward (Kortsch et al., 2015) and vertical (John & Post, 2021) migrations) and decreased vulnerability, i.e. the number of predator species per prey (Snickars et al., 2015). Additionally, the Baltic Sea is particularly prone to eutrophication due to its long water retention time and large historical inputs of nitrogen and phosphorus (Andersen et al., 2017), which have been found to simplify benthic trophic networks by reducing number of taxa, links, and chain length, while increasing connectance (Nordström & Bonsdorff, 2017; O'Gorman et al., 2012).

While anthropogenic pressures are known to affect biodiversity and ecosystem functions in the Baltic Sea, it is unknown how these changes are cascading through the ecosystem and which functions could be altered by additional pressures. Anthropogenic pressures are likely to affect the Baltic Sea basins disproportionately, affecting benthic communities, trophic networks, and ecosystem functions in varying magnitudes (Griffiths et al., 2017). Benthic trophic networks, with important ecosystem‐based management implications, have not been continuously monitored and we do not know how their structural changes have led to functional changes in the Baltic Sea (EU MSFD, 2008; Olivier et al., 2019; Rogers et al., 2010; Tam et al., 2017). The low species richness of the Baltic Sea have been well studied (Snoeijs‐Leijonmalm et al., 2017), and feeding interactions thoroughly documented (see Janas et al., 2017, and references therein), with the exception of the fully marine Skagerrak (where the Baltic Sea meets the North Sea), where biodiversity and feeding links among the taxa there have not been investigated to the same degree.

In this study, we aim to evaluate changes in the benthic invertebrate food webs of the Swedish coast, utilizing long‐term monitoring data and literature dietary links to model highly resolved food webs in a stressed ecosystem. We explore how environmental gradients and combined anthropogenic pressures have affected the benthic trophic network architecture, assessing changes in a broad suite of well‐established, complementary metrics, as well as through changes in species richness and community composition during the study period. In particular, we expect food web structure to vary along the salinity gradient of the Baltic Sea, with higher salinities correlating with higher trophic network complexity due to the co‐occurring species richness gradient. We also predict that changes in food web structure over time will be more evident on the Swedish west coast than for other basins due to large species loss during the study period (Obst et al., 2018).

## 2 MATERIALS AND METHODS

### 2.1 Study area

**FIGURE 1:**
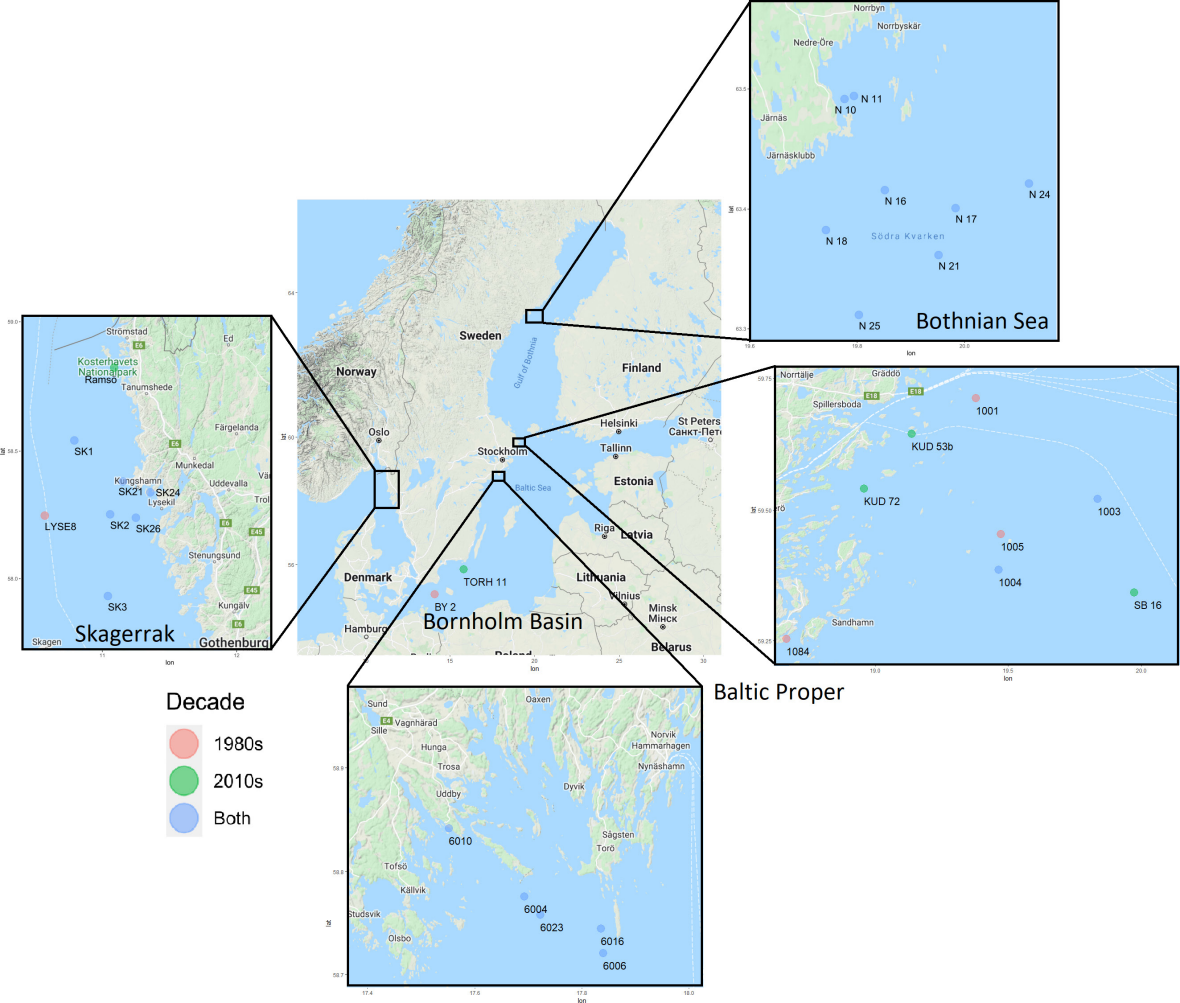
Map of paired benthic monitoring stations used in this study. Stations in blue were present in both decades, in pink only in the 1980s, and in green only in the 2010s. Note that the stations present in only the 1980s (pink) and only 2010s (green) were then paired for the analysis based on similar physical characteristics (see Methods)

The Swedish national benthic invertebrate monitoring program has been continuously sampling Baltic Sea benthic invertebrates since the 1980s once per year in May. We chose to investigate food webs in 1980–1989, when benthic monitoring was conducted over a large geographical region using standardized methods for the first time, and 2010–2019, as the most recent decade of gathered data. The 26 included stations cover most of the Swedish coast of the Baltic Sea, from the almost freshwater conditions in the north of the Bothnian Sea (average salinity 5.8) to the marine conditions of the Skagerrak (average salinity 34), where the Baltic Sea meets the North Sea (Figure 1). Benthic organisms were sampled with a van Veen sediment grab (0.1 m^2^) and sieved through 1 mm mesh (Broman et al., 2019). When more than one replicate was taken, organism abundance was averaged over the replicates to reduce heterogeneity between sampling effort in the basins. Organisms were preserved, sorted, counted, and weighted in the lab according to the European standard, and identified to the lowest possible taxonomic classification (82% to species level, 11% to genus level, and 7% to family or order level; hereafter referred to as "species" for simplicity; EN 16665:2014, 1992). Taxa composition and abundance data were retrieved from the Swedish Meteorological and Hydrological Institute's database for marine monitoring data, Sharkweb (sharkweb.smhi.se). Data from Sharkweb (the Baltic Proper, Bornholm Basin, and Skagerrak) were complemented with additional regional monitoring data from the Bothnian Sea collected by Umeå Marine Sciences Centre using the same methods previously described.

For this study, stations were selected from monitoring datasets if present in a majority of years, or if similar stations existed, as judged by physical characteristics (depth and salinity), as well as geographical closeness (average distance 67 km between paired non‐identical stations). As fewer stations were present in the 1980s dataset than the 2010s dataset, we searched the 2010s dataset for stations with similar depth and salinity to 1980s stations (when the exact same stations were not present in both datasets). If multiple 2010s stations matched the 1980s depth and salinity, we selected the stations geographically closest to the 1980 station. Only stations below the photic zone (below 20 m in the Baltic Sea) were selected to allow for control of food sources (i.e., no photosynthesis production), and only soft sediment habitats were selected. The depths of stations analyzed range from 20.4 to 301 m, with a mean of 59.8 m. Salinity ranges from 4 to 34, with a mean of 7.4. Abiotic data can be found in Figure S1. Four basins, bodies of the Baltic Sea separated by shallow sills, were considered here by where our stations were located. Each basin has distinct characteristics, including variation in physical characteristics, such as salinity and temperature, and biological characteristics, such as species richness. Twenty‐six stations (or pairs of stations) were included in the final dataset. Eight stations are included in the Bothnian Sea, 10 stations in the Baltic Proper, 1 station in the Bornholm Basin, and 7 in the Skagerrak (Figure 1). For ease of comparison, stations are referred to by the 1985 name in the text when names differ. Data are available from the Dryad Digital Repository: https://doi.org/10.5061/dryad.bk3j9kdfk, and sampling effort can be found in Table S1.

### 2.2 Literature review, food web construction, and metrics evaluated

A literature review of feeding ecology of all taxa found in our dataset was conducted to determine their respective trophic links. Feeding links were only used when peer‐reviewed sources showed feeding either from direct observation, feeding trials, or gut content analysis (Nordström et al., 2015; Nordström & Bonsdorff, 2017). References span the years 1937 to 2020, and Baltic Sea area studies were preferentially selected when possible, but when those were not available, references from Europe were taken. We did not impose a minimum number of individuals used to determine the diet of a given predator. Feeding link matrices and literature sources can be found in the Dryad Digital Repository: https://doi.org/10.5061/dryad.bk3j9kdfk. We included two basal resources (phytoplankton and detritus) as nodes, although we recognize that selection within these resources is occurring. The benthic invertebrate monitoring program unfortunately does not record information about composition or biomass of phytoplankton and detritus. Five rare taxa were excluded from the analysis as no feeding information could be found or inferred from the available literature (the gastropod family *Diaphanidae*, gastropod species *Mangelia attenuate* and *Taranis moerchii*, and the annelid species *Drilonereis filum* and *Glyphonesione klatti*). Additionally, if there was uncertainty that the taxonomic resolution would interfere with the integrity of the trophic network construction, then the node was removed (e.g., all recordings of the class “Hydrozoa” were removed from Skagerrak databases due to the large diet diversity of Hydrozoa, but species‐level observations were kept).

A metaweb was assembled for each basin of the study, consisting of all taxa present in all years and all possible feeding links (Figure S2). From each basin metaweb, individual food webs were constructed for each station and year. While each basin has its own metaweb, we did not adjust the metawebs for spatial or temporal variation, meaning that links were assumed to be consistent across basins and decades when species co‐occurred. This approach is conservative in estimating species interactions based on species composition changes and removes allowances for rewiring, as no *in situ* documentation could be provided. The package “igraph” in R (Csardi & Nepusz, 2006; R Core Team, 2021) was used for food web assembly and metric calculations, along with additional functions (trophic level and omnivory) from Kortsch et al. (2021). To identify changes in food web structure, we selected 12 complementary and widely used food web metrics in order to describe whole‐network properties, including vertical and horizontal dimensions of the food web across strong gradients (taxonomic richness and salinity) and time. The selected metrics include species richness (*S*), number of links (*L*), linkage density (*Z*), connectance (*C*), generality (*G*), vulnerability (*V*), mean distance (*D*), shortest path (*P*), trophic level (*T*), omnivory (*O*), number of motifs (*M*), and modularity (*Q*). The food web metrics were calculated for each station web in each year, and their calculations and ecological meaning are described in Table 1. Note that for simplicity, linkage density, mean distance, number of motifs, and modularity are presented in the Table S2.

**TABLE 1:** Food web metrics utilized in this study, formulas, definitions, and ecological implications

Metric	Formula	Definition and references	Ecological implications
Species richness (S)		Number of species present in the web	Biodiversity measurement
Number of links (*L*)		Number of links present in the web	Number of energy pathways in a community, with implications for the complexity of the food web
Linkage density (*Z*)	$\frac{L}{S}$	Number of interactions per species (Dunne et al., 2002)	Gives an estimate of how connected species are, on average, within a food web
Connectance (*C*)	$\frac{L}{S^{2}}$	Proportion of realized interactions from the total possible interactions (Dunne et al., 2002)	Indicates robustness or resistance to change
Generality (*G*)	$\frac{\sum_{k=1}^{j}a_{\mathrm{jk}}}{n_{j}}$	Mean number of prey per predator (Schoener, 1989)	Relative bias toward generalist or specialist predators; lower values indicate more specialist predators
Vulnerability (*V*)	$\frac{\sum_{k=1}^{i}a_{\mathrm{kj}}}{n_{i}}$	Mean number of predators per prey (Schoener, 1989)	Relative risk of becoming prey; lower values indicate less predators
Mean distance (*D*)	$\frac{\sum d_{i,j}}{S}$, where $d_{i,j}=\left(x_{\mathrm{ih}}+\cdots +x_{\mathrm{hj}}\right)$ and *h* is the intermediate species between species *i* and *j*	Average of the number of links between any two given species in the network (Williams et al., 2002)	Higher values indicate longer paths and less efficient energy transfer
Mean shortest path (*P*)	$\frac{\sum d_{i,j}}{S}$, where $d_{i,j}=\min\left(x_{\mathrm{ih}}+\cdots +x_{\mathrm{hj}}\right)$ and *h* is the intermediate species between species *i* and *j*	Average of the shortest path between any two given species in the network (Kortsch et al., 2021)	Indicative of stability, shorter paths are more stable than long ones
Mean trophic level (*T*)	$\frac{\sum_{i}T_{i}}{S}$	Short‐weighted trophic levels, combination between shortest trophic level and prey‐averaged trophic level (Kortsch et al., 2021)	Related to complexity, but also more trophic levels result in less efficient energy transfer
Proportion of omnivory (*O*)	$O_{i}=\sqrt{\frac{\sum_{j}{\left(T_{j}‐T_{i}\right)}^{2}}{Din_{i}}}$, then averaged for the whole food web:$O=\frac{\sum_{i}O_{i}}{S}$	Proportion of species in the network that feed on multiple trophic levels (Kortsch et al., 2021)	Omnivores are stabilizing to food webs due to their high linkage and ability to prey switch
Number of motifs (*M*)		Total number of three‐species connected subgraphs in the network (Bascompte & Melián, 2005)	More motifs indicate a more complex network; structure underling basic interactions
Modularity (*Q*)	$\frac{1}{2L}\sum_{i,j}\left(A_{\mathrm{ij}}‐\frac{k_{i}k_{j}}{2L}\right)\delta \left(c_{i},c_{j}\right)$	Number of more interconnected groups of species in the network (Clauset et al., 2004; Grilli et al., 2016)	Compares if groups (“modules”) are more or less connected than random aggregations of species; indicates structure of network, with more modules being more stable

### 2.3 Statistical analysis

Data visualization was conducted in R using the “ggplot2” and “ggmap” packages (Kahle & Wickham, 2013; Wickham, 2016). All statistical tests were run in R (v4.1.2; R Core Team, 2021). Permutational multivariate analysis of variance (PERMANOVA) tests to detect differences in food web metrics were run using basin, decade, and station nested within basin as independent variables and each food web metric as a response variable separately. The adonis2 function in the “vegan” package was used, with 999 permutations utilizing Euclidean distance, and pairwise t‐tests were used to determine differences between basins and decade using the Holm adjustment method for multiple comparisons (Oksanen et al., 2019). Unpaired Welch t‐tests were conducted between the decades for the paired stations after checking for assumptions. P values were adjusted for multiple comparisons by the Holm adjustment method. Non‐metric multidimensional scaling (NMDS) was run using Bray–Curtis similarity on community composition for the different basins and decades in the “vegan” package. An additional PERMANOVA using the same function to 999 permutations, but Bray–Curtis distance, was run to test significant differences in abundances of community composition with decade, basin, and their interaction as independent variables. A SIMPER (similarity percentages) analysis was run using the “vegan” package within each basin to detect significant drivers of community differences. Finally, in order to visualize correlation between the food web metrics investigated here, a correlation analysis was run with “corrplot” (Figure S3; Wei & Simko, 2021). A principal component analysis (PCA) was also run to identify the main spatial (basin) and temporal (decade) dynamics in food web structure, as well as assess complementarity among metrics. The package “ggbiplot” was utilized to visualize the PCA (Vu, 2011).

## 3 RESULTS

### 3.1 Basin metawebs

The Bothnian Sea dataset had 19 species total (all years) and increased in species richness from 12 in the 1980s to 16 in the 2010s. There were three unique species in the 1980s (16%) and seven unique species in the 2010s (37%), with nine species found in both decades (47%). The Bothnian Sea metaweb had 57 links, a linkage density of 3, and a connectance of 0.16 (i.e., 16% of all possible trophic interactions are realized). The Baltic Proper had a total of 27 species in the dataset, and increased from 23 to 25 species during the study period. Twenty‐one species, or 78%, were found in both years, and 2 (7%) and 4 (15%) were unique to the 1980s and 2010s, respectively. A total of 82 links were identified in the metaweb, resulting in a linkage density of 3.04 and a connectance of 0.11. In the Bornholm Basin, a total of 31 species were found, and decreased from 22 in the 1980s to 18 in the 2010s. Thirteen species were unique to the 1980s (42%), nine were unique to the 2010s (29%), and nine were found in both decades (29%). The number of links in the Bornholm Basin metaweb was 102, resulting in a linkage density of 3.29 and a connectance of 0.11. A significant species loss occurred within the study period for the Skagerrak (*t*‐test *t* = 13, *p *< .001). A total of 381 species were present in the dataset, decreasing from 336 species in the 1980s to 244 in the 2010s. There were 137 unique species in the 1980s dataset (40%), only 45 unique to the 2010s dataset (12%), and 199 present in both decades (52%). The metaweb had a total of 10,647 links, a linkage density of 28, and a connectance of 0.073.

### 3.2 Basin comparison in food web metrics

**FIGURE 2:**
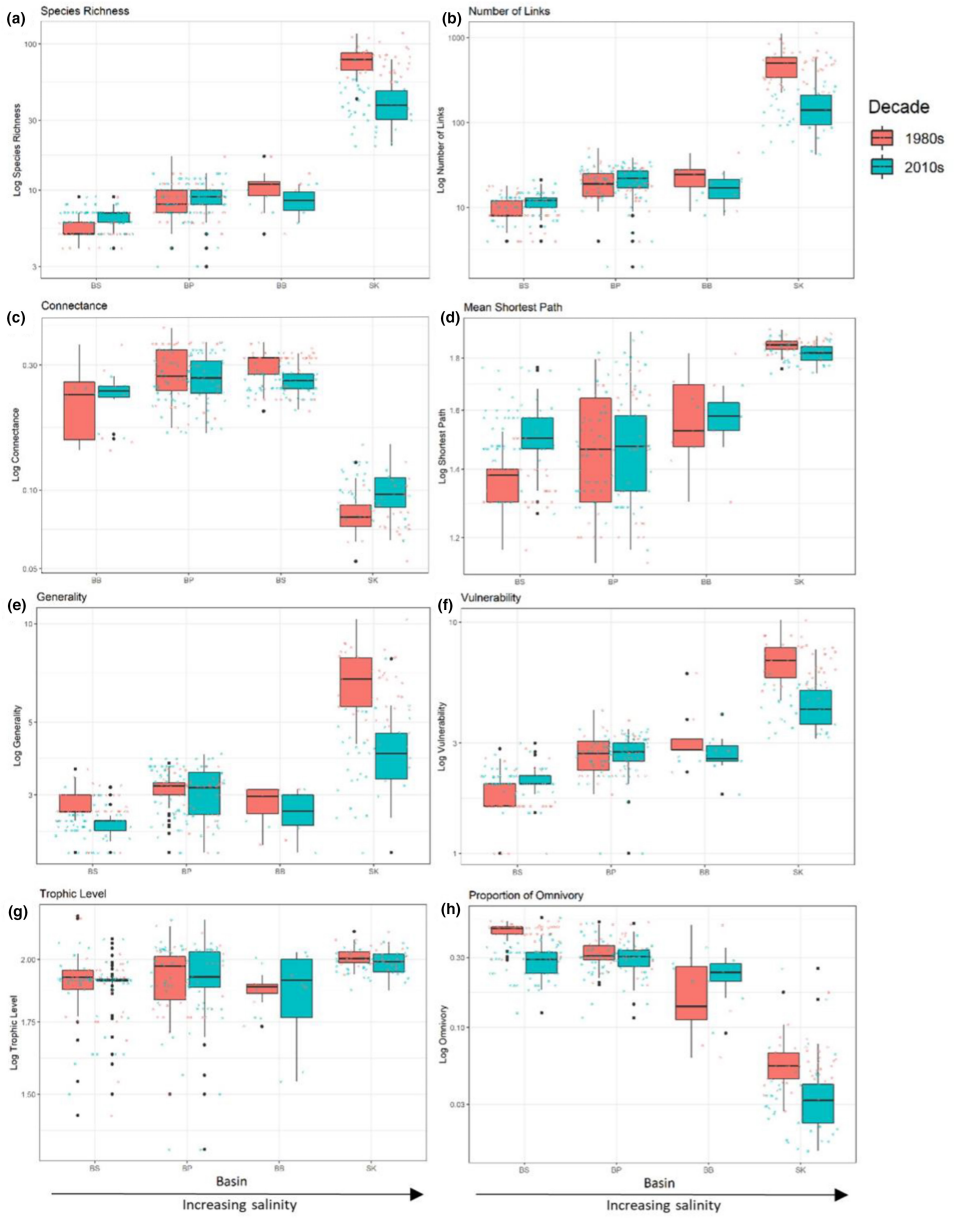
Changes in (a) species richness, (b) number of food web links, (c) food web connectance, (d) mean shortest path, (e) generality of predator diet, (f) vulnerability, or the number of predators per prey, (g) mean trophic level, and (h) omnivory, for different basins of the Baltic Sea in the 1980s (red) and 2010s (turquoise). *Y*‐axes are log transformed to best visualize the data. Basins: BS, Bothnian Sea; BP, Baltic Proper; BB, Bornholm Basin; SK, Skagerrak

**FIGURE 3:**
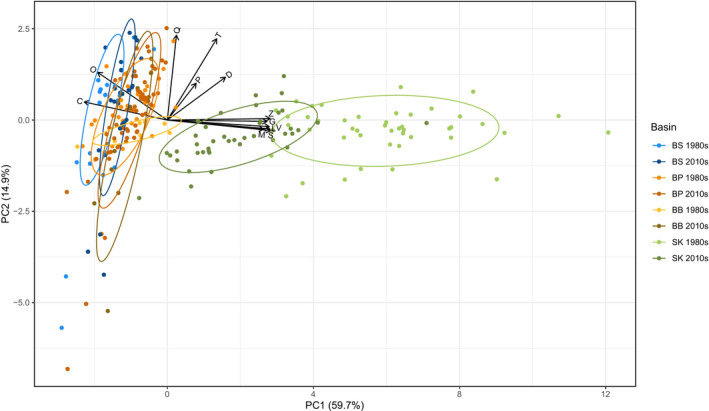
Principle component analysis (PCA) of the 12 food web metrics investigated here in the different basins and two decades, with the combined two axes explaining 74.6% of the variation in metrics. Food web metrics: *S*, species richness; *L*, number of links; *Z*, linkage density; *C*, connectance; *G*, generality; *V*, vulnerability; *D*, mean distance; *P*, mean shortest path; *T*, mean trophic level; *O*, omnivory; *M*, number of motifs; *Q*, modularity. Basins: BS, Bothnian Sea; BP, Baltic Proper; BB, Bornholm Basin; SK, Skagerrak

There were significant differences between the four basins for all food web metrics. Species richness (*S*), number of links (*L*), generality (*G*), vulnerability (*V*), and mean shortest path (*P*) were all highest at the Skagerrak basin when compared to Bothnian Sea, Baltic Proper, and Bornholm Basin, indicating higher food web complexity with higher salinity (PERMANOVA, *S*: *F*_3,359_ = 1478, *p *= .001; *L*: *F*_3,359_ = 525, *p *= .001; *G*: *F*_3,359_ = 405, *p *= .001; *V*: *F*_3,358_ = 705, *p *= .001; *P*: *F*_3,359_ = 206, *p *= .001; Table 2, Figure 2). Additionally, Skagerrak had significantly higher trophic level (*T*) than the Bothnian Sea and Baltic Proper (*T*: *F*_3,359_ = 25, *p *= .001), although not the Bornholm Basin. However, connectance (*C*) and proportion of omnivory (*O*) showed the opposite pattern, being significantly lower in the Skagerrak compared with the other basins (PERMANOVA, *C*: *F*_3,359_ = 551, *p *= .001; *O*: *F*_3,359_ = 375, *p *= .001; Table 2, Figures 2 and 3). This higher food web complexity in more saline basins was further indicated by significantly higher generality and vulnerability in the Baltic Proper than in Bothnian Sea food webs (PERMANOVA, *G*: *F*_3,359_ = 405, *p *= .001; *V*: *F*_3,358_ = 705, *p *= .001; Table 2, Figure 2). The Bornholm Basin also showed significantly higher vulnerability than the Bothnian Sea (PERMANOVA, *V*: *F*_3,358_ = 705, *p *= .001). Connectance and omnivory continued to show inverse trends with salinity, with the Bothnian Sea and Baltic Proper showing significantly higher connectance than the Bornholm Basin (PERMANOVA, *C*: *F*_3,359_ = 551, *p *= .001; *O*: *F*_3,359_ = 375, *p *= .001; Table 2, Figure 2). Station (nested within basin) was a significant explanatory variable for all metrics (PERMANOVA, *S*: *F*_27,359_ = 4.14, *p *= .02; *L*: *F*_27,359_ = 4.22, *p *= .001; *C*: *F*_27,359_ = 3.93, *p *= .001; *G*: *F*_27,359_ = 3.76, *p *= .001; *V*: *F*_27,358_ = 4.49, *p *= .001; *P*: *F*_27,338_ = 4.47, *p *= .001; *T*: *F*_27,359_ = 6.66, *p *= .001; and *O*: *F*_27,359_ = 4.17, *p *= .001; Table 2). Additional metrics can be found in Figure S4 and Table S3.

**TABLE 2:** Permutational multivariate analysis of variance (PERMANOVA) model results with basin, decade, station nested within basin, and an interaction effect between basin and decade as independent variables and food web metrics for the response variable, tested individually

Response variable	General model					
	Independent variable	SS	df	*R* ^2^	*F*	*p*
*S*	Basin	176,217	3	.78	1478	.**001**
	Decade	7559	1	.033	190	.**001**
	Station(Basin)	4441	27	.02	4.14	.**02**
	Basin × Decade	25,529	3	.11	214	.**001**
*L*	Basin	6,912,633	3	.59	525	.**001**
	Decade	654,071	1	.056	149	.**001**
	Station(Basin)	499,697	27	.043	4.22	.**001**
	Basin × Decade	2,027,334	3	.17	154	.**001**
*C*	Basin	2.24	3	.77	551	.**001**
	Decade	0.02	1	.007	15	.**002**
	Station(Basin)	0.14	27	.049	3.93	.**001**
	Basin × Decade	0.045	3	.015	11	.**001**
*G*	Basin	455	3	.58	405	.**001**
	Decade	51	1	.066	137	.**001**
	Station(Basin)	38	27	.049	3.76	.**001**
	Basin × Decade	101	3	.13	90	.**001**
*V*	Basin	725	3	.71	705	.**001**
	Decade	23	1	.023	68	.**001**
	Station(Basin)	42	27	.041	4.49	.**001**
	Basin × Decade	108	3	.11	105	.**001**
*P*	Basin	7.89	3	.56	206	.**001**
	Decade	0.2	1	.014	16	.**001**
	Station(Basin)	1.54	27	.11	4.47	.**001**
	Basin × Decade	0.46	3	.033	12	.**001**
*T*	Basin	0.67	3	.13	25	.**001**
	Decade	0.0091	1	.0017	1.03	.32
	Station(Basin)	1.59	27	.3	6.66	.**001**
	Basin × Decade	0.0088	3	.0016	0.33	.79
*O*	Basin	5.32	3	.64	375	.**001**
	Decade	0.46	1	.056	97	.**001**
	Station(Basin)	0.53	27	.064	4.17	.**001**
	Basin × Decade	0.42	3	.051	30	.**001**

Response variable	Pairwise *t*‐test comparisons						
	BS vs. BP	BS vs. BB	BS vs. SK	BP vs. BB	BP vs. SK	BB vs. SK	1980s vs. 2010s
*S*	0.25	0.53	**<0.001**	0.8	**<0.001**	**<0.001**	**<0.001**
*L*	1	1	**<0.001**	1	**<0.001**	**<0.001**	**<0.001**
*C*	0.83	**<0.001**	**<0.001**	**<0.001**	**<0.001**	**<0.001**	0.98
*G*	**<0.001**	0.52	**<0.001**	0.27	**<0.001**	**<0.001**	**<0.001**
*V*	**<0.001**	**<0.001**	**<0.001**	0.28	**<0.001**	**<0.001**	**<0.001**
*P*	0.32	**0.011**	**<0.001**	**0.027**	**<0.001**	**<0.001**	0.35
*T*	0.14	0.203	**<0.001**	0.086	**<0.001**	**<0.001**	0.21
*O*	**<0.001**	**<0.001**	**<0.001**	**<0.001**	**<0.001**	**<0.001**	**0.0017**

Note

Pairwise *t*‐test comparisons with Holm *p*‐value correction results are below. Significant values (*p* < .05) are indicated in bold. Response food web variables: *S*, species richness; *L*, number of links; *C*, connectance; *G*, generality; *V*, vulnerability; *P*, mean shortest path; *T*, mean trophic level, and *O*, omnivory. Basins: BS, Bothnian Sea; BP, Baltic Proper; BB, Bornholm Basin; SK, Skagerrak.

The PCA analysis shows the variability in food web metrics in different Baltic basins (Figure 3), with the PC1 axis accounting for approximately 60% of variability and PC2 for 15%. The Bothnian Sea, Baltic Proper, and Bornholm Basins cluster together, while the Skagerrak clusters separately, mainly driven by differences in species richness, number of links, linkage density, generality, vulnerability, and number of motifs (Figure 3).

### 3.3 Temporal changes

Together with basins, decade was also a significant explanatory factor for all food web metrics, with the exception of trophic level, indicating that a significant shift in food web complexity has occurred between the 1980s and 2010s in all basins (PERMANOVA, *S*: *F*_1,359_ = 190, *p *= .001; *L*: *F*_1,359_ = 149, *p* = .001; *C*: *F*_1,359_ = 15, *p* = .002; *G*: *F*_1,359_ = 137, *p* = .001; *V*: *F*_1,358_ = 68, *p* = .001; *P*: *F*_1,338_ = 16, *p* = .001; and *O*: *F*_1,359_ = 97, *p* = .001; Table 2, Figure 2). However, there was a significant interactive effect between decade and basin for all metrics, with the exception of trophic level (PERMANOVA, *S*: *F*_3,359_ = 214, *p* = .001; *L*: *F*_3,359_ = 154, *p* = .001; *C*: *F*_3,359_ = 11, *p* = .001; *G*: *F*_3,359_ = 90, *p* = .001; *V*: *F*_3,358_ = 105, *p* = .001; *P*: *F*_1,338_ = 12, *p* = .001; *O*: *F*_3,359_ = 30, *p* = .001; Table 2), which indicates that the effect of decade was not consistent throughout all the basins. Additional metrics can be found in Figure S4 and Table S3.

The PCA also revealed a different clustering between the two decades for the Skagerrak region, suggesting that this basin changed the most between 1980s and 2010s, mainly driven by reduction in species richness, number of links, linkage density, generality, vulnerability, and number of motifs (Figure 3).

### 3.4 Station‐level changes

Results from the unpaired Welch *t*‐tests on the station level between the decades are listed in Table S3 and visualized in Figure S5. In the Bothnian Sea, three stations of eight significantly increased in species richness between the time period studied here (*S*, Welch *t*‐test N11: *t* = −3.7, *p*_adj_ = .04; N17: *t* = −4.9, *p*_adj_ = .007; and N21: *t* = −5, *p*_adj_ = .005). Only two showed corresponding significantly increased number of links (*L*, N11: *t* = −4.1, *p*_adj_ = .02; N21: *t* = −4.7, *p*_adj_ = .01). Three stations demonstrated significantly decreased connectance from the 1980s to the 2010s (*C*, N16: *t* = 6.8, *p*_adj_ = .0007; N24: *t* = 10.8, *p*_adj_ = .00004; and N25: *t* = 6.2, *p*_adj_ = .002). Five stations showed significantly decreased generality (*G*, N10: *t* = 1.4, *p*_adj_ = .01; N11: *t* = 3.6, *p*_adj_ = .05; N16: *t* = 4.6, *p*_adj_ = .01; N24: *t* = 4.1, *p*_adj_ = .02; and N25: *t* = 4.7, *p*_adj_ = .02). Conversely, only one station showed significantly increased vulnerability (*V*, N21 *t* = −0.1, *p*_adj_ = .003). Additionally, one station increased shortest path (*P*, N11: *t* = −6.1, *p*_adj_ = .006). Five stations showed significantly decreased proportion of omnivory (*O*, N16: *t* = 7.7, *p*_adj_ = .002; N17: *t* = 7.2, *p*_adj_ = .0003; N18: *t* = 6.7, *p*_adj_ = .0009; N24: *t* = 10.3, *p*_adj_ = .0003; N25: *t* = 6.2, *p*_adj_ = .001), potentially decreasing food web stability. All stations in the Bothnian Sea changed significantly in at least one metric.

In the Baltic Proper, no stations changed significantly in terms of species richness (*S*) from the 1980s to the 2010s. One station showed a significant increase in number of links (6004, *L t* = −6.6, *p*_adj_ = .0003). One station significantly decreased in connectance (*C*, 6010 *t* = 3.9, *p*_adj_ = .05). The Bornholm Basin station did not change significantly for any food web metrics between the 1980s and the 2010s.

All seven Skagerrak stations showed a significant decline in species richness (*S*, SK3: *t* = 6.02, *p*_adj_ = .007; SK26: *t* = 4.5, *p*_adj_ = .02; LYSE8: *t* = 4.5, *p*_adj_ = .02; SK2: *t* = 6.8, *p*_adj_ = .001; SK24: *t* = 5.5, *p*_adj_ = .005; SK21: *t* = 7.1, *p*_adj_ = .0004; and SK1: *t* = 5.4, *p*_adj_ = .006), and six stations demonstrated subsequent significant number of links decreases (*L*, SK3: *t* = 7.7, *p*_adj_ = .02; LYSE8: *t* = 4.4, *p*_adj_ = .03; SK2: *t* = 5.4, *p*_adj_ = .03; SK24: *t* = 5.7, *p*_adj_ = .008; SK21: *t* = 5.6, *p*_adj_ = .007; and SK1: *t* = 4.7, *p*_adj_ = .03). No Skagerrak stations changed significantly in terms of connectance (*C*). Four stations showed significant declines in both generality (*G*, SK3: *t* = 6.7, *p*_adj_ = .007; SK2: *t* = 5.8, *p*_adj_ = .051; SK21: *t* = 4.1, *p*_adj_ = .03; and SK1: *t* = 4.5, *p*_adj_ = .02) and vulnerability (*V*, SK3: *t* = 7.4, *p*_adj_ = .001; SK2: *t* = 6.1, *p*_adj_ = .006; SK21: *t* = 4.2, *p*_adj_ = .03; and SK1: *t* = 4.9, *p*_adj_ = .01).

### 3.5 Macrofauna community composition

PERMANOVA results showed significant differences in macrofauna community composition between basins (*F*_3,0.31_ = 70.8, *p* = .001), with the NMDS indicating clustering based on our defined basins (NMDS, stress = 0.102; Figure S6). In particular, the Skagerrak communities clustered separately from the other basins. PERMANOVA results further indicated a significant difference in community composition between decades (*F*_1,0.077_ = 52.5, *p* = .001), and a significant interaction between decade and basin (*F*_3,0.095_ = 21.5, *p* = .001). SIMPER analysis indicated that the community changes between decades in the Bothnian Sea were driven primarily by the disappearance of *Bylgides sarsi*, and decrease in absolute abundance of *Cyanophthalma obscura*, *Monoporeia affinis*, and *Mysidae*. Changes in the Baltic Proper were driven by the decrease in *Bathyporeia pilosa*, *Bylgides sarsi*, *Monoporeia affinis*, and Oligochaeta, as well as the disappearance of *Theodoxus fluviatilis* and appearance of Nemertea. Bornholm Basin community changes were driven by the appearance of *Cyanophthalma obscura*, Nemertea, and *Saduria entomon*, and the disappearance of Oligochaeta. *Marenzelleria* spp. also appeared in all three basins during the study period. In the Skagerrak, 239 species changed significantly in terms of abundance during the study period (Table S4).

## 4 DISCUSSION

Benthic invertebrate food webs have changed significantly from the 1980s to the 2010s in our study area, generally decreasing in species richness, number of links, linkage density, and motifs, and becoming less complex in terms of generality of predators, vulnerability of prey, proportion of omnivorous species, and modularity. Our results indicate that most of this change happened in the Skagerrak, where benthic food webs were reorganized to a simpler network architecture. The Skagerrak basin was also significantly different from the other basins in all food web metrics except for modularity, indicating network complexity in our study was positively correlated with salinity. This is furthered by the significantly higher linkage density, generality, and vulnerability in Baltic Proper food webs compared to the lower saline basin of the Bothnian Sea. This study focuses on large structural food web changes in the macrozoobenthos that have received little attention in the Baltic Sea, one of the best‐studied large marine ecosystems, highlighting the need to include macrozoobenthos interactions into management decisions. For example, Niiranen et al. (2012) found that macrozoobenthos exhibited strong bottom‐up control on cod when modeling commercial fish productivity under future climate scenarios, but was one of the most variable components of the model due to scarce data and high spatial variability. While monitoring biodiversity and biomass is useful, monitoring trophic interactions through well‐studied metrics provides some missing puzzle pieces, supplying more information about changes in ecosystem function. With better understanding of interactions and drivers within the macrozoobenthos, such as those provided by the present study, commercial fishing could be better modeled with climate scenarios.

Our results indicate that food webs in the higher salinity basin Skagerrak were more affected than the less saline basins, and it is likely that species adapted to the physiological stresses of the Baltic Sea brackish water environment are also resilient to additional correlated pressures, such as pH fluctuations and high seasonality (Rousi et al., 2019). We recognize that salinity is correlated with a number of other factors in the Baltic Sea (species richness and temperature), and the positive network complexity–salinity relationship seen here will not be necessarily true in all systems. For example, in the Mediterranean Sea, pelagic species richness is inversely correlated with salinity, with consequences for pelagic food web complexity (Piroddi et al., 2015). Alternatively, the higher diversity of the marine Skagerrak includes taxa that are potentially more susceptible to novel pressures, as suggested by the large drop in biodiversity in our dataset. The levels of anthropogenic pressures vary with basin (Viitasalo et al., 2015), and thus give some implications for stress interactions and which communities are susceptible to which pressures.

### 4.1 Anthropogenic pressures and food web changes

The species richness loss found in the Skagerrak is comparable to previously reported 51.9% reduction in species richness over 88 years from the same area (Obst et al., 2018). Obst et al. (2018) found similar numbers of species (their 254 to our 244) in the 2000s, but found a total of 607 species were present between 1920 and 1940, considerably higher than the 336 species from the 1980s found here. As sampling effort rarefaction curves indicate undersaturation in species richness in the earlier samplings of 1920–1940 (Obst et al., 2018), it is possible that macrozoobenthos decrease in species richness and impact in food web metrics is in fact even higher than what is indicated by our data. The current study differs from previous studies in that we investigate how species loss impacts trophic networks.

Previous studies proposed bottom trawling fishing and increase in turbidity as main drivers of macrozoobenthos biodiversity loss in the Skagerrak (Obst et al., 2018). Most bottom trawling occurs in the Skagerrak and Bornholm Basin for demersal fish species (mostly cod *Gadus morhua*; ICES, 2019), although there is a prawn fishery (*Pandalus borealis*) in the Skagerrak, but this species was not present in our dataset (Linders et al., 2018). Around 80 to 100% of the seabed is effected by bottom trawling in the Skagerrak and Bornholm Basin (HELCOM, 2018a), despite declines in cod catch per unit effort since the peak in the 1980s (Möllmann et al., 2021). In addition to physical disturbance, bottom trawling can also resuspend contaminants, and large stores of persistent organic pollutants exist in the soft sediments of the Baltic Sea (Jonsson, 2000). Such contaminants disproportionately affect larger and long‐lived species (Bradshaw et al., 2012). Predators are generally larger and longer lived than their prey (Nordström et al., 2015), and we would expect to see reduced vulnerability and trophic level if contaminants due to bottom trawling were underlying the observed biodiversity loss. However, predator proportions in our Skagerrak trophic networks increased from 18.5% to 22.3% from the 1980s to 2010s, and there was no significant change in trophic level. Interestingly, we saw significant decreases in vulnerability despite higher proportions of predators, mainly in the Baltic Proper and Skagerrak regions, indicating an increase in specialist predators or a decrease in prey diversity. Increased turbidity primarily negatively affects suspension feeders (Bock & Miller, 1995; Sañé et al., 2013), but there was an increase in proportion of suspension feeders in the Skagerrak from the 1980s to the 2010s (from 13.1% to 14.3%, but overall decrease in species richness (from 43 to 34 species)), demonstrating that suspension feeders were not more impacted than other trophic groups during the study period. As such, bottom trawling is not likely to be the sole explanation for reduced species richness in the Skagerrak basin.

In addition to decreased species richness, we observed an increase in connectance in Skagerrak trophic networks. O'Gorman et al. (2012) found similar increased connectance when multiple stressors were present, associated with a decrease in species richness and proportion of top predators. They postulated that replacement of specialist top predators with generalists increases realized links while reducing top predator diversity. Responses to multiple stressors showed higher ecosystem function variability, indicating greater volatility over time (O'Gorman et al., 2012). While the Skagerrak connectance in our study increased, generality decreased and proportion of top predators increased (but total numbers of predators decreased), disagreeing with the findings of O'Gorman et al. (2012). We did find a small increase in proportion of predatory species that are generalists (from 86.9 to 90.6%; generalist predators defined here as those feeding on multiple taxonomic groups of prey), while seeing an overall decrease in number of predators (from 61 to 53 species) and number of generalist predators (from 53 to 48 species). This small shift toward a larger proportion of generalist predators could be an effect of multiple pressures, as has been found in O'Gorman et al. (2012), but our definition of generalist predators is arbitrary and we did not see a definitive shift toward generalist predators. The increase in connectance with simultaneous declines in generality, vulnerability, and omnivory could be indicative of vertical network compression, and we observed a non‐significant trend of declining mean trophic level (Figure 2g) in the Skagerrak, including declines at all stations except one (Figure S5i). Vertical network compression has been reported in Baltic Sea (Kortsch et al., 2021) and North Sea (Frelat et al., 2022) trophic networks previously.

Vulnerability decreased in the Bornholm Basin and Skagerrak, indicating prey are less vulnerable to predation by other benthic invertebrates. One explanation is an increase in specialist predators; another is that loss of prey items is driving decreased generality in the Bornholm Basin and Skagerrak. Conversely, the Bothnian Sea showed increased vulnerability, contrary to other basins, while generality decreased. We hypothesize that this increased vulnerability while generality decreased is due to increased importance of specialist predators that interact with a subset of prey, while increasing the overall number of predators that each prey is consumed by. Indeed, the significant decline in proportion of omnivory at most stations in the Bothnian Sea (Figure S5j and Table S3) supports the specialization of predators, and increased proportion of predators has been reported in the Bothnian Sea before (Törnroos et al., 2019). However, only one guild (benthic invertebrates) was investigated in this study, and predation from other guilds should be considered in future research.

In the Bothnian Sea and Baltic Proper, increases in taxa richness indicate local species invasions during the study period, but these invasions, while increasing the dispersion, did not significantly change the means of most trophic network metrics, which suggests that the invasions had little effect on the benthic invertebrate trophic network architecture here investigated. However, rates of species introductions in marine ecosystems are increasing (Hulme, 2009), and monitoring for consequences on trophic networks should continue (Ojaveer et al., 2017).

Eutrophication is known to simplify food webs through fewer nodes, links, shorter chains, and increased connectance (Nordström & Bonsdorff, 2017; O'Gorman et al., 2012). Eutrophication affects all basins studied here, although with different magnitudes. The 5‐point HELCOM eutrophication status report from 1985 to 2011–2016 shows improvement from moderate to good–moderate status in the Bothnian Sea, retained moderate–poor in the Baltic Proper, retained bad in the Bornholm Basin, and improved from moderate to good–moderate in the Kattegat (close to the Skagerrak, which was not evaluated) (Andersen et al., 2017; HELCOM, 2018b). However, our study indicates that changes in eutrophication status did not produce effects on Baltic Proper, Bornholm Basin, and Skagerrak trophic networks, contradicting previous studies (Nordström & Bonsdorff, 2017; O'Gorman et al., 2012; Pearson & Rosenberg, 1978). For example, a clear simplification of Skagerrak trophic networks was evident in our study despite the eutrophication status in this basin remaining the same or improving during the study period. Nevertheless, the improvement in Bothnian Sea eutrophication status could be tied to the maintenance of trophic networks. It is important to note that hypoxic stations were excluded from this study and the benthic monitoring program due to lack of organisms, and thus, connections between our results and trends in eutrophication status of the study areas could not be easily evaluated, as in other studies (Vaquer‐Sunyer & Duarte, 2011). Hypoxia is one of the strongest abiotic factors structuring benthic communities in the Baltic Sea (Conley et al., 2009). Studies found that overall Baltic Sea trophic networks have deteriorated, despite improvement in general Baltic Sea eutrophication status from poor to moderate status from 1985 to 2015, due to a reduction in nutrient inputs (Andersen et al., 2017; Murray et al., 2019). While nutrient inputs to the Baltic Sea have been reduced over the past few decades, the hypoxia‐affected area of the Baltic Sea continues to increase, largely due to rising temperatures, and has caused large functional changes in the last 40 years (Carstensen & Conley, 2019; Pecuchet et al., 2020). The basin most affected by hypoxia, the Baltic Proper, did not show cohesive significant changes in the trophic network metrics from our stations. Habitat compression will be of concern in the future, when Baltic Proper species are constrained from poleward migrations by land masses and low salinity, and prevented from migration to deeper waters by hypoxia. Additionally, benthic communities in the 1980s are likely already impacted by eutrophication in the Baltic Sea (Karlson et al., 2002; Pearson & Rosenberg, 1978), and earlier reference periods would be needed to evaluate food web metrics from communities unaffected by eutrophication. However, the benthic monitoring program did not begin to regularly sample until the 1980s, and thus, evaluating changes before eutrophication began to impact the Baltic Sea is difficult.

Climate change and eutrophication interact to produce large‐scale impacts on trophic networks (Gilarranz et al., 2016). Climate change may nullify nutrient reductions and increase hypoxic zones in the Baltic due to temperature rise (Reusch et al., 2018). Temperature and hypoxia can act synergistically on benthic organisms; heat stress decreases organism tolerance of hypoxic conditions, creating further community composition and trophic network changes that manifest in reduced species richness, number of links, generality, vulnerability, and number of motifs (Vaquer‐Sunyer & Duarte, 2011). The Baltic Proper and Bothnian Sea are more vulnerable to hypoxia than other basins due to longer water residence times and more pronounced stratification (Conley et al., 2009), and the low species richness indicates that any local extinctions will bring severe impacts for trophic network functioning. While we did not observe the potential network effects of temperature and eutrophication in these basins, the interaction of local, regional, and global pressures should be further investigated, as these have been shown to cause both food web simplification and functional reorganization in the Baltic (Pecuchet et al., 2020; Törnroos et al., 2019). Climate change has been connected to increased connectance, which could be behind the changes seen in the Skagerrak, and increased variability of generality, which we could see evidence of in Figure 2e (Kortsch et al., 2019), but more studies focusing on these variables with more complete abiotic data will be required to make definite conclusions.

Of course, there are limitations with this study. Long‐term monitoring datasets, while valuable, come with difficulties of standardization and shifting methods (Magurran et al., 2010). The monitoring program samples once per year in May and could have missed species present at different times of the year. Thus, we were not able to consider the seasonal differences in food webs. In addition, only considering two decades of monitoring data could also skew our findings, as we may miss shifts in community composition. We also focus only on benthic invertebrate macrofauna, and it would be beneficial to expand the study to include lower trophic levels, such as meiofauna, as well as higher trophic levels. We assume feeding links from literature, and this information is incomplete due to the stochastic nature of species interactions, and will likely overestimate the number of links (Roslin & Majaneva, 2016). In addition, we were not able to quantify the strength of the feeding links (Kortsch et al., 2021) or present high resolution of basal resources. We hope that future studies will consider these limitations and aid in better trophic network resolution. Nevertheless, we were able to show changed trophic networks utilizing existing long‐term monitoring data, which has important management implications.

### 4.2 Management suggestions and conclusions

We found a simplification of modeled Baltic Sea macrozoobenthic trophic networks during the period 1980 to 2019, and the Skagerrak region was most affected, in agreement with our predictions. We also detected changes in benthic invertebrate food web structures that were not related to taxonomic richness, underlying the importance of not only monitoring benthic communities but also their interactions through metrics to understand and predict changes in ecosystem functions. Commercially important fish are likely to depend on benthic invertebrates in aphotic habitats, and decreasing benthic invertebrate biomass is a concern that there may be trophic cascades resulting in decreased fish stocks in the future (Snickars et al., 2015). Conservation of benthic invertebrate trophic networks is thus essential to maintain valuable ecosystem functions. Other studies of anthropogenic pressures on whole ecosystem marine food webs have found that marine‐protected areas improve food web resilience (Gilarranz et al., 2016). We recommend further studies investigating whether expanding and adding Swedish marine protected areas, particularly in the Skagerrak, would reduce anthropogenic pressures and conserve benthic invertebrate trophic networks.

## AUTHOR CONTRIBUTIONS

**Julie A. Garrison:** Conceptualization (equal); Data curation (lead); Formal analysis (lead); Methodology (lead); Writing – original draft (lead); Writing – review & editing (equal). **Marie C. Nordström:** Methodology (equal); Writing – review & editing (equal). **Jan Albertsson:** Data curation (equal); Writing – review & editing (equal). **Francisco J. A. Nascimento:** Conceptualization (equal); Funding acquisition (lead); Writing – review & editing (equal).

## CONFLICT OF INTEREST

The authors have no conflict of interest to declare.

## Supporting information

Supplementary Material

## Data Availability

The data is available at the Dryad Digital Repository: https://doi.org/10.5061/dryad.bk3j9kdfk.
