# Supplementary material for: Temporal and spatial changes in benthic invertebrate trophic networks along a taxonomic richness gradient
Source: Ecol Evol. 2022 Jun 5;12(6):e8975. doi: 10.1002/ece3.8975 (PMC9168554; doi:10.1002/ece3.8975)
Supplement: Supplementary file 1 — Supplementary Material [file ECE3-12-e8975-s001.docx]

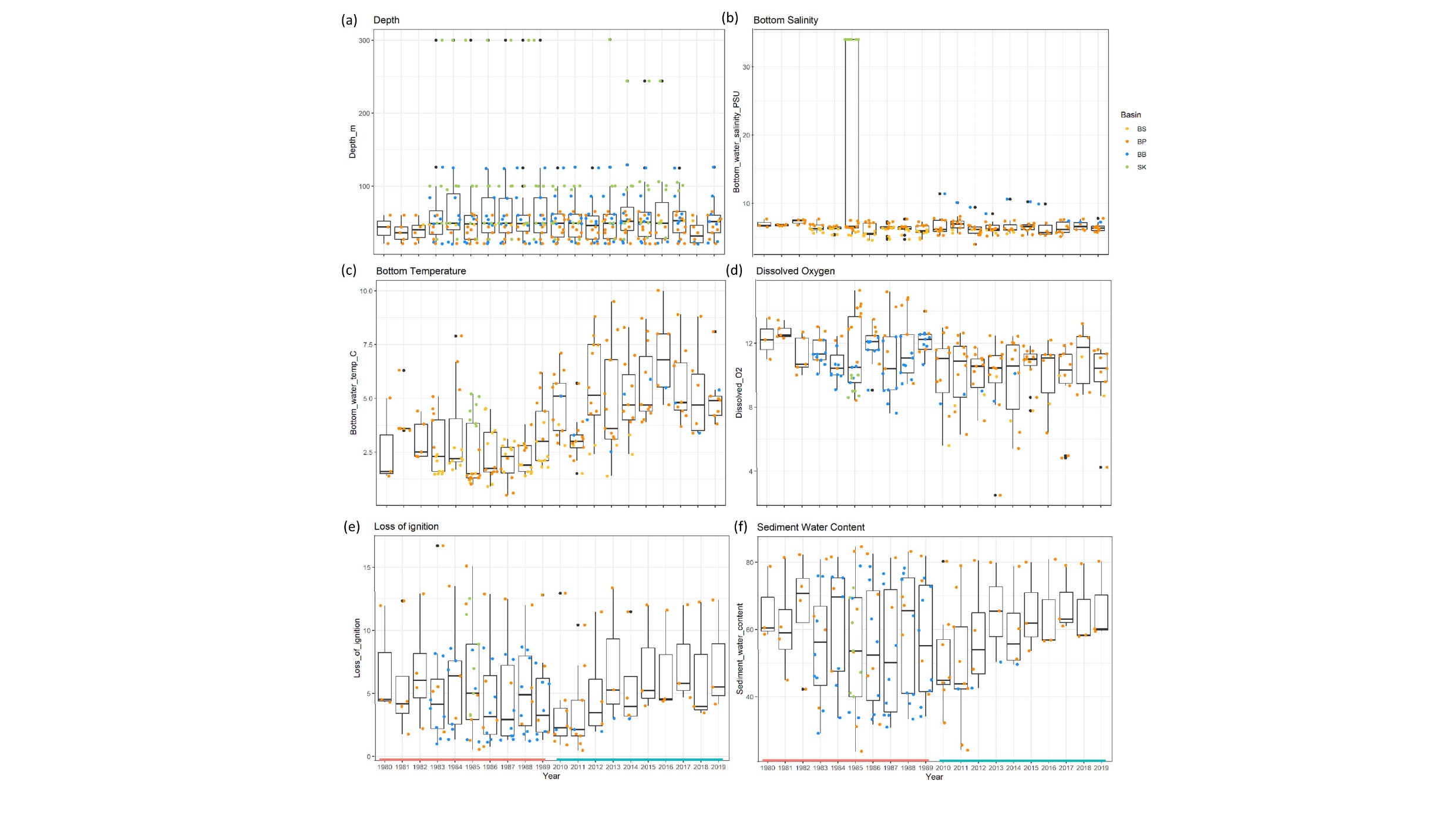
Figure S1. Abiotic characteristics of stations where food web metrics were evaluated in the 1980’s (red line) and 2010’s (turquoise line) for a) depth in meters, b) bottom water salinity in practical salinity units, c) bottom water temperature in °C, d) bottom water dissolved oxygen in l/l, e) sediment loss of ignition of C in percent dry weight, and f) sediment water weight in percent wet weight. Points represent individual stations in the Bornholm Basin (BB, yellow), Baltic Proper (BP, orange), Bothnian Sea (BS, blue) or Skagerrak (SK, green) basins of the Baltic Sea. Note that data was not available for all stations in all years; salinity is missing 147 values, temperature 149 values, dissolved oxygen 152 values, loss of ignition 222 values, and sediment water content 222 values.


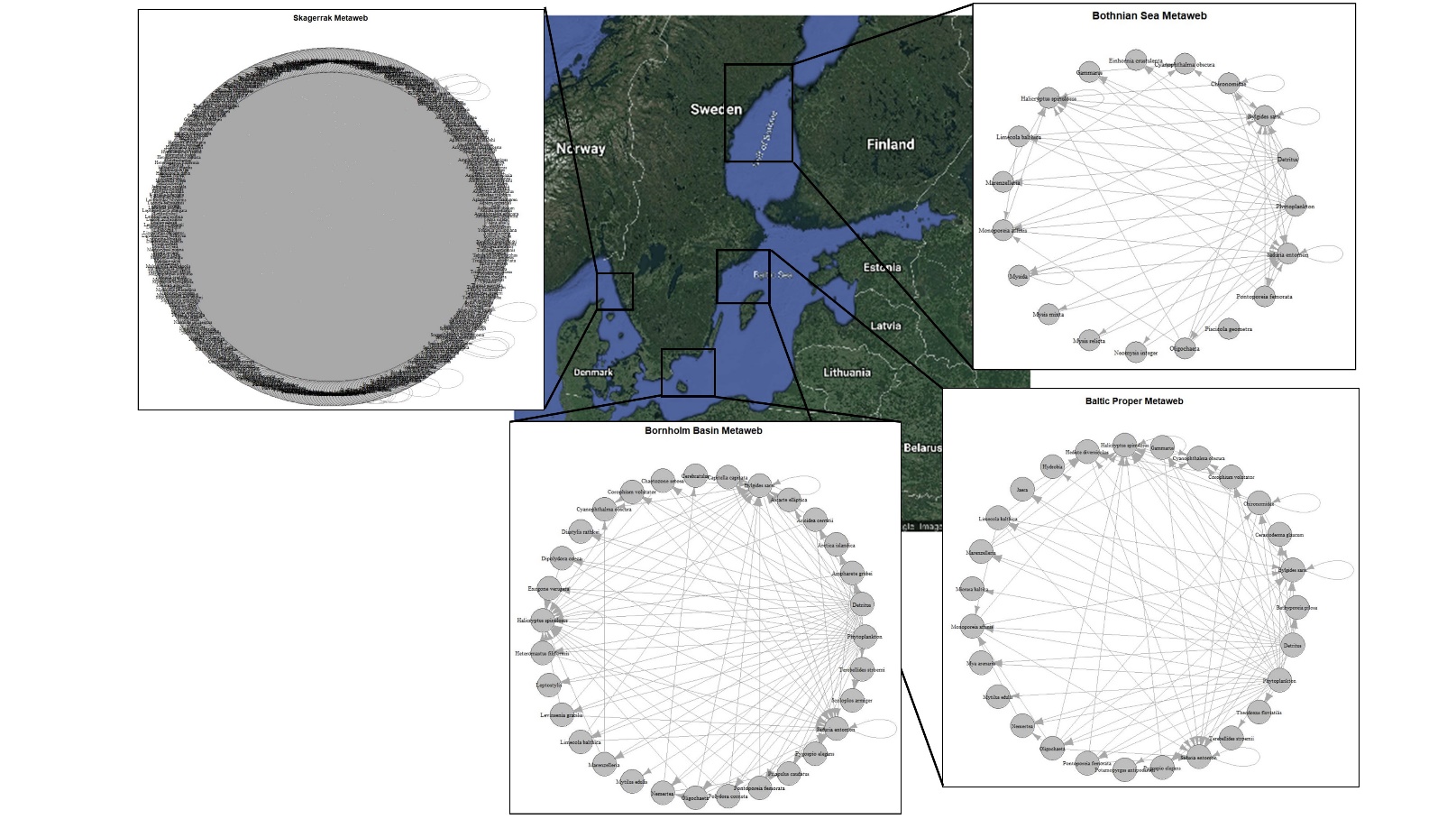


Figure S2. Trophic networks of all taxa present in the dataset and all links (“metaweb”) for each basin (Skagerrak, Bornholm Basin, Baltic Proper and Bothnian Sea) for the study period. Taxa are represented by grey circles, and directed feeding links from the prey to the predator are represented by arrows.


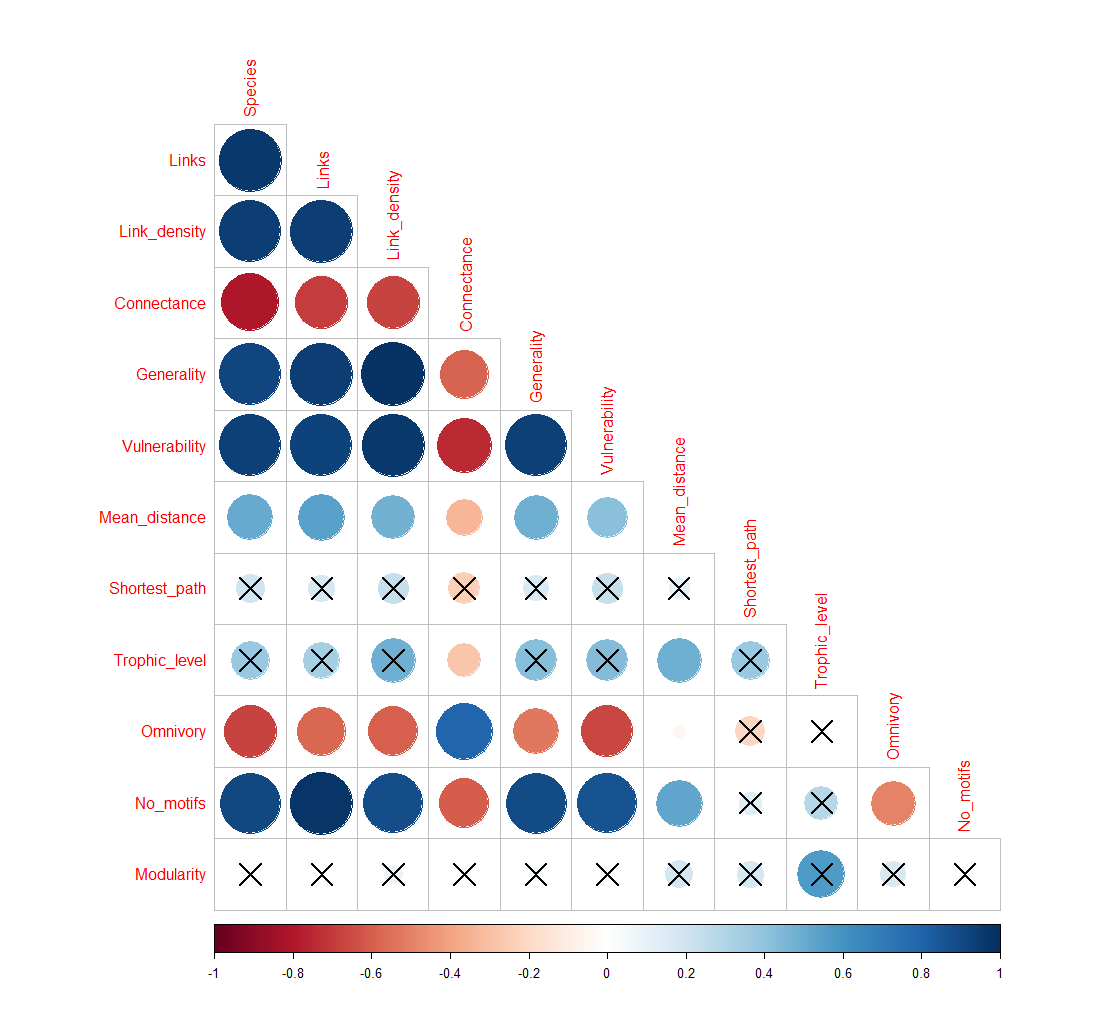


Figure S3. Pearson Product-Moment correlations between food web metrics, where the colors indicate the direction and strength of the correlation, the size of the circles represent the strength of the correlation, and x indicates that the correlation is not significant (*p*>0.05).


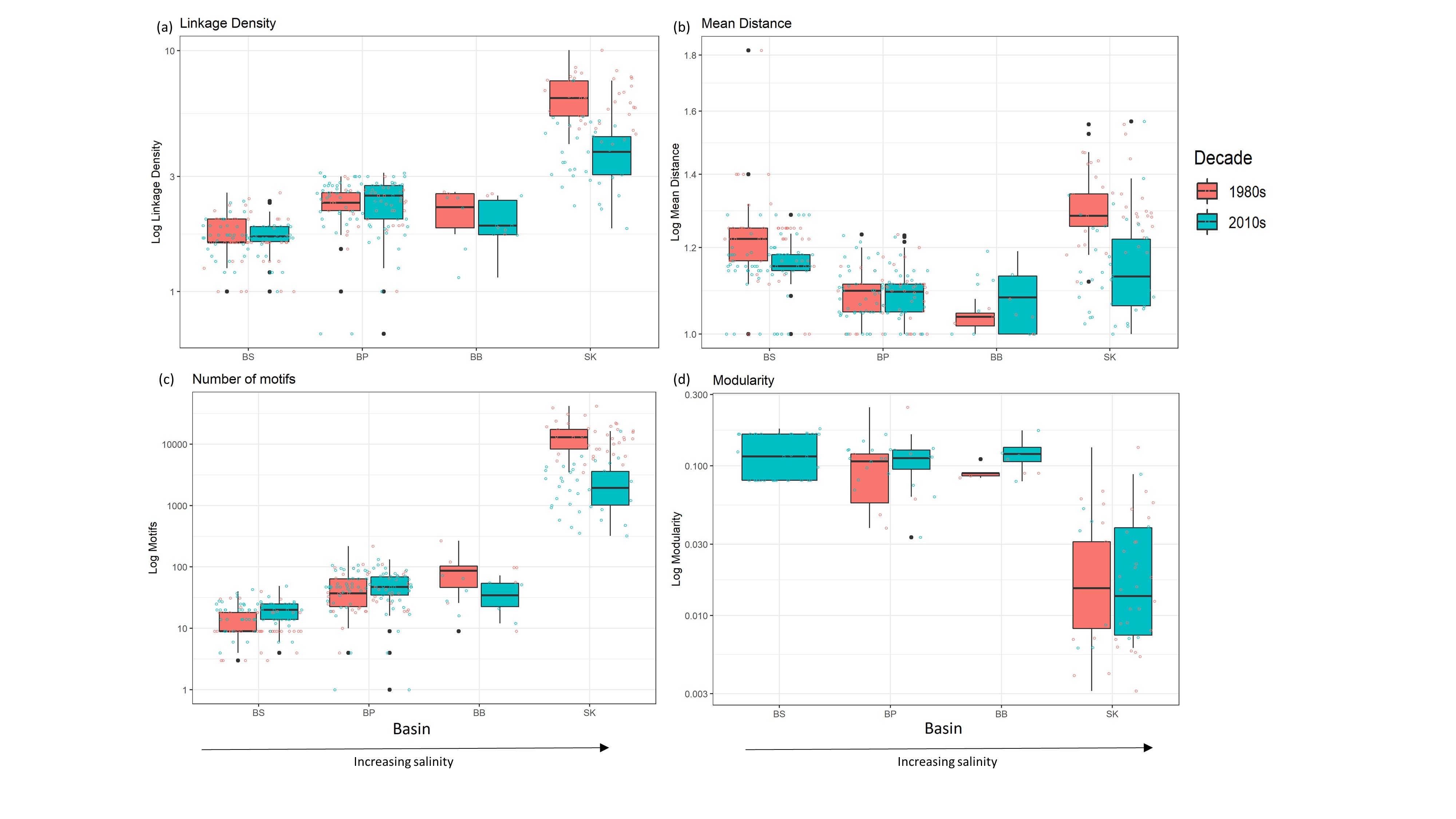


Figure S4. Changes in a) food web linkage density, b) mean distance, c) number of motifs, and d) modularity for different basins of the Baltic Sea in the 1980’s (red) and 2010’s (turquoise). Y-axes are log transformed to best visualize the data. Basins: BS Bothnian Sea; BP Baltic Proper; BB Bornholm Basin; SK Skagerrak.


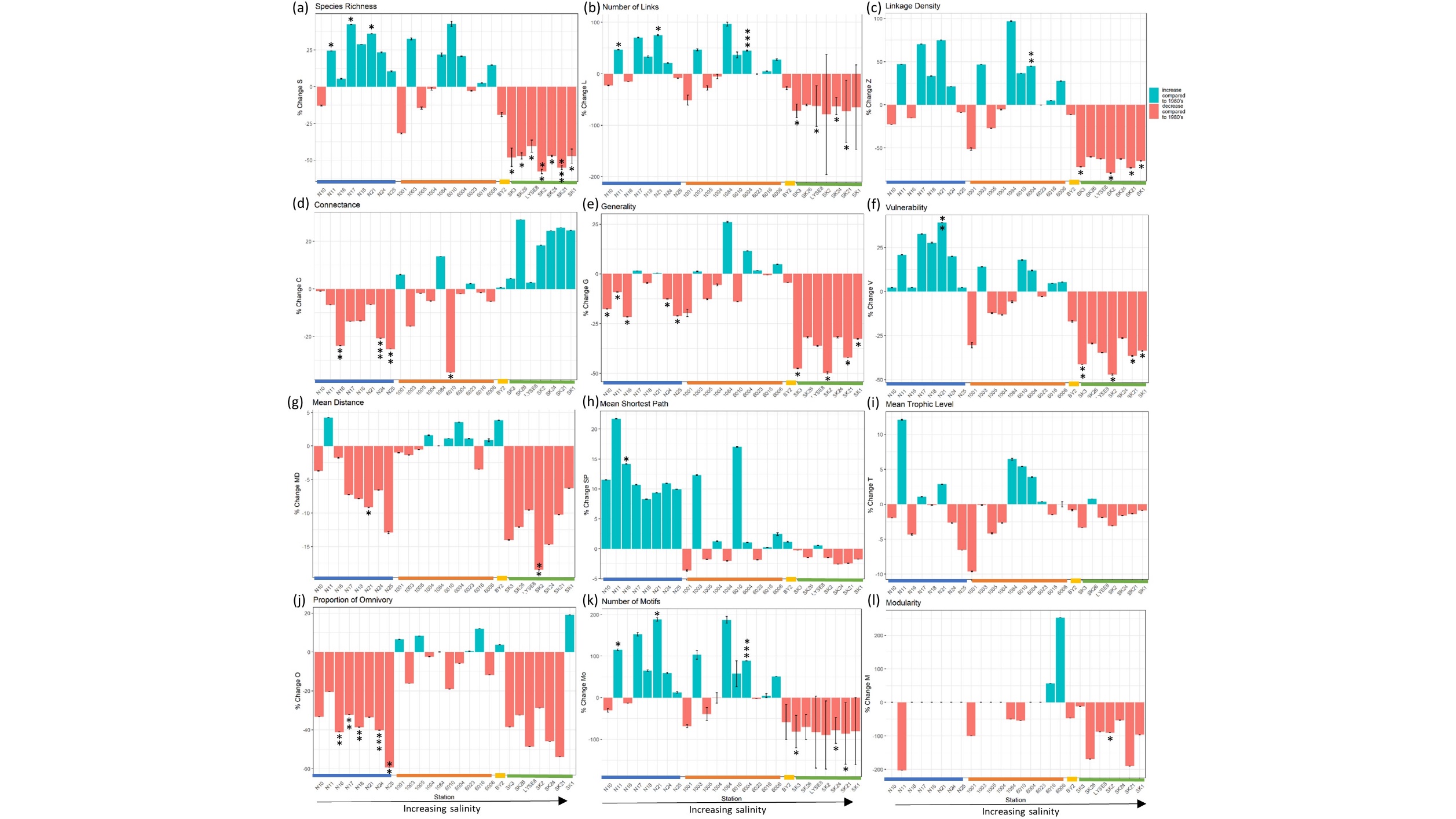


Figure S5. Percent changes in paired stations between the 1980’s and 2010’s in a) species richness, b) number of food web links, c) food web linkage density, d) food web connectance, e) generality of predator diet, f) vulnerability, or the number of predators per prey, g) mean distance, h) mean shortest path, i) mean trophic level, j) omnivory, k) number of motifs, and l) modularity with standard deviation. Red indicates a decrease in the food web metric and turquoise indicates an increase. Welch t-test with Holm *p* adjustment for multiple comparisons demonstrate significant difference between the decades, with * indicating *p*<0.05, ** indicating *p*<0.005, and *** indicating *p*<0.0005. Blue bar BS Bothnian Sea; Orange bar BP Baltic Proper; Yellow bar BB Bornholm Basin; Green bar SK Skagerrak. Stations are arranged in order of increasing salinity.


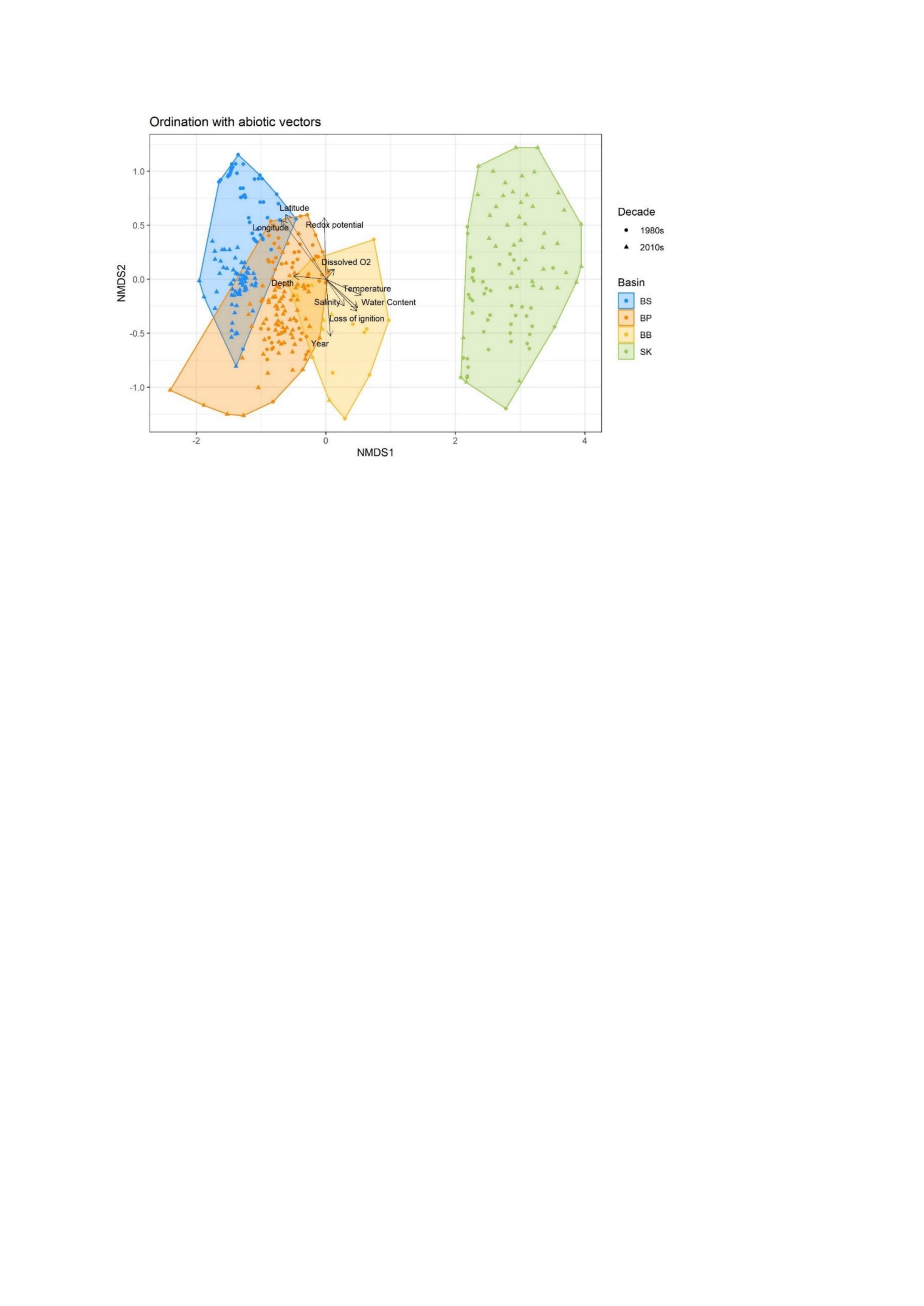


Figure S6. Non-metric multidimensional scaling (nMDS) of benthic invertebrate communities in the different basins of the Baltic Sea in the 1980’s (circles) and 2010’s (triangles) with arrows representing abiotic variables. Stress = 0.102. Basin in order of increasing salinity: BS Bothnian Sea, BP Baltic Proper, BB Bornholm Basin, SK Skagerrak.

Table S1. Number of networks present in the dataset by year, decade, and basin. Note than stations present in multiple years have been counted for each year. BS Bothnian Sea, BP Baltic Proper, BB Bornholm Basin, SK Skagerrak.

| Basin | 1980 | 1981 | 1982 | 1983 | 1984 | 1985 | 1986 | 1987 | 1988 | 1989 | 1980’s |
| --- | --- | --- | --- | --- | --- | --- | --- | --- | --- | --- | --- |
| BS | 0 | 0 | 0 | 8 | 8 | 4 | 8 | 8 | 8 | 8 | 52 |
| BP | 3 | 5 | 5 | 5 | 3 | 10 | 5 | 5 | 5 | 5 | 51 |
| BB | 0 | 0 | 1 | 1 | 1 | 1 | 1 | 1 | 1 | 1 | 8 |
| SK | 0 | 0 | 0 | 6 | 7 | 7 | 7 | 7 | 4 | 7 | 45 |
| Total | 3 | 5 | 6 | 20 | 19 | 22 | 21 | 21 | 18 | 21 | 156 |
|  | **2010** | **2011** | **2012** | **2013** | **2014** | **2015** | **2016** | **2017** | **2018** | **2019** | **2010’s** |
| BS | 8 | 8 | 8 | 8 | 8 | 8 | 2 | 8 | 2 | 8 | 68 |
| BP | 10 | 10 | 9 | 10 | 8 | 10 | 5 | 9 | 5 | 8 | 84 |
| BB | 1 | 1 | 1 | 1 | 1 | 1 | 1 | 1 | 1 | 1 | 10 |
| SK | 6 | 6 | 0 | 6 | 7 | 7 | 7 | 3 | 0 | 0 | 42 |
| Total | 25 | 25 | 18 | 25 | 24 | 26 | 15 | 21 | 8 | 17 | 204 |

Table S2. Permutational multivariate analysis of variance (PERMANOVA) model results with basin, decade, station nested within basin, and an interaction effect between basin and decade as independent variables and food web metrics for the response variable, tested individually. Pairwise t-test comparisons with Holm p-value correction results are below. Significant values (*p* < 0.05) are indicated in bold. Response food web variables: Z linkage density, D mean distance, M number of motifs, and Q modularity. Basins: BS Bothnian Sea, BP Baltic Proper, BB Bornholm Basin, SK Skagerrak.

|  | | General model | | | | | | | | | | | |
| --- | --- | --- | --- | --- | --- | --- | --- | --- | --- | --- | --- | --- | --- |
| Response variable | | Independent variable | | SS | | | df | R^2^ | | F | | | *p* |
| Z | | Basin | | 649 | | | 3 | 0.67 | | 612 | | | **0.001** |
|  |  | Decade | | 35 | | | 1 | 0.036 | | 98 | | | **0.001** |
|  |  | Station(Basin) | | 45 | | | 27 | 0.047 | | 4.72 | | | **0.001** |
|  |  | Basin x Decade | | 109 | | | 3 | 0.11 | | 103 | | | **0.001** |
| D | | Basin | | 1.45 | | | 3 | 0.31 | | 75 | | | **0.001** |
|  |  | Decade | | 0.3 | | | 1 | 0.065 | | 46 | | | **0.001** |
|  |  | Station(Basin) | | 0.35 | | | 27 | 0.075 | | 1.99 | | | **0.011** |
|  |  | Basin x Decade | | 0.35 | | | 3 | 0.075 | | 18 | | | **0.001** |
| M | | Basin | | 5059500000 | | | 3 | 0.44 | | 222 | | | **0.001** |
|  |  | Decade | | 759290000 | | | 1 | 0.066 | | 100 | | | **0.001** |
|  |  | Station(Basin) | | 671740000 | | | 27 | 0.058 | | 3.27 | | | **0.004** |
|  |  | Basin x Decade | | 2297200000 | | | 3 | 0.2 | | 101 | | | **0.001** |
| Q | | Basin | | 0.018 | | | 3 | 0.0088 | | 1.27 | | | 0.27 |
|  |  | Decade | | 0.016 | | | 1 | 0.0079 | | 3.44 | | | 0.071 |
|  |  | Station(Basin) | | 0.38 | | | 27 | 0.18 | | 2.97 | | | **0.002** |
|  |  | Basin x Decade | | 0.095 | | | 3 | 0.046 | | 6.63 | | | **0.001** |
|  | Pairwise t-test comparisons | | | | | | | | | | | | |
| Response variable | BS vs. BP | | BS vs. BB | | BS vs. SK | BP vs. BB | | | BP vs. SK | | BB vs. SK | 1980’s vs. 2010’s | |
| Z | **<0.001** | | 0.23 | | **<0.001** | 0.24 | | | **<0.001** | | **<0.001** | **<0.001** | |
| D | **<0.001** | | **<0.001** | | **0.0018** | 0.21 | | | **<0.001** | | **<0.001** | **<0.001** | |
| M | 1 | | 1 | | **<0.001** | 1 | | | **<0.001** | | **<0.001** | **<0.001** | |
| Q | 1 | | 1 | | 1 | 0.79 | | | 1 | | 0.79 | 0.094 | |

Table S3. Unpaired Welch t-tests between food web metrics in the 1980’s and the 2010’s in Baltic Sea stations, with *p* values adjusted by the Holm method for multiple comparisons. Significant values (*p* < 0.05) are in bold. Stations are placed in order of increasing salinity. Basin: BS Bothnian Sea, BP Baltic Proper, BB Bornholm Basin, SK Skagerrak. Food web metrics: S species richness, L number of links, Z linkage density, C connectance, G generality, V vulnerability, D mean distance, P mean shortest path, T mean trophic level, O omnivory, M number of motifs, Q modularity.

|  |  | S | | L | | Z | | C | | G | | V | |
| --- | --- | --- | --- | --- | --- | --- | --- | --- | --- | --- | --- | --- | --- |
| Basin | Station | t statistic | Adj *p*-value | t statistic | Adj *p*-value | t statistic | Adj *p*-value | t statistic | Adj *p*-value | t statistic | Adj *p*-value | t statistic | Adj *p*-value |
| BS | N10 | 1.4 | 1 | 1.4 | 1 | 1.3 | 1 | 0.13 | 1 | 4.3 | **0.012** | -0.22 | 1 |
| BS | N11 | -3.7 | **0.035** | -4.1 | **0.016** | -2.4 | 0.42 | 0.82 | 1 | 3.6 | **0.046** | -3.1 | 1 |
| BS | N16 | -1.1 | 1 | 1.5 | 1 | 3.2 | 0.13 | 6.8 | **0.00073** | 4.6 | **0.012** | -0.4 | 1 |
| BS | N17 | -4.9 | **0.0068** | -3.5 | 0.053 | -2.1 | 0.65 | 2.4 | 0.52 | -0.23 | 1 | -3.7 | 0.13 |
| BS | N18 | -2.7 | 0.17 | -1.5 | 1 | -0.61 | 1 | 2.4 | 0.58 | 0.61 | 1 | -1.9 | 0.93 |
| BS | N21 | -5.0 | **0.0052** | -4.7 | **0.013** | -3.4 | 0.089 | 0.95 | 1 | -0.09 | 1 | -6.0 | **0.0034** |
| BS | N24 | -3.3 | 0.081 | -1.6 | 1 | 0.11 | 1 | 10.8 | **0.000045** | 4.1 | **0.022** | -3.1 | 0.13 |
| BS | N25 | -1.2 | 1 | 0.63 | 1 | 2.6 | 0.36 | 6.2 | **0.0018** | 4.7 | **0.017** | -0.26 | 1 |
| BP | Norrtalje | 0.43 | 1 | 0.57 | 1 | 0.43 | 1 | 0.14 | 1 | 0.46 | 1 | 1.3 | 1 |
| BP | 6010 | -3.3 | 0.081 | -1.9 | 0.87 | 0.085 | 1 | 3.9 | **0.049** | 2.5 | 0.28 | -2.3 | 0.41 |
| BP | 6004 | -3.2 | 0.067 | -6.6 | **0.00028** | -4.9 | **0.0029** | 0.27 | 1 | -2.3 | 0.301 | -2.8 | 0.2002 |
| BP | 6023 | 0.28 | 1 | 0.0094 | 1 | -0.206 | 1 | -0.35 | 1 | -0.29 | 1 | 0.37 | 1 |
| BP | 6016 | -0.26 | 1 | -0.29 | 1 | -0.22 | 1 | 0.21 | 1 | 0.079 | 1 | -0.6 | 1 |
| BP | 6006 | -2.1 | 0.45 | -2.7 | 0.099 | -1.7 | 1 | 0.66 | 1 | -0.802 | 1 | -1.03 | 1 |
| BB | BY2 | 1.5 | 1 | 1.6 | 1 | 1.3 | 1 | -0.046 | 1 | 0.64 | 1 | 1.2 | 1 |
| SK | SK3 | 6.02 | **0.0074** | 7.7 | **0.02** | 6.6 | **0.0065** | -0.50 | 1 | 6.7 | **0.0066** | 7.4 | **0.00108** |
| SK | SK26 | 4.5 | **0.019** | 3.3 | 0.094 | 2.5 | 0.42 | -3.1 | 0.18 | 2.6 | 0.29 | 2.7 | 0.27 |
| SK | LYSE8 | 4.5 | **0.022** | 4.4 | **0.028** | 3.6 | 0.20 | -0.25 | 1 | 3.3 | 0.26 | 4.1 | 0.108 |
| SK | SK2 | 6.8 | **0.00103** | 5.4 | 0.28 | 6.03 | **0.0086** | -2.03 | 0.89 | 5.8 | **0.012** | 6.06 | **0.006** |
| SK | SK24 | 5.5 | **0.0052** | 5.7 | **0.008** | 3.3 | 0.20 | -1.4 | 1 | 3.1 | 0.20 | 3.6 | 0.13 |
| SK | SK21 | 7.1 | **0.00046** | 5.5 | **0.0071** | 4.02 | **0.041** | -2.4 | 0.58 | 4.06 | **0.031** | 4.2 | **0.033** |
| SK | SK1 | 5.4 | **0.0063** | 4.7 | 0.28 | 4.7 | **0.013** | -2.5 | 0.49 | 4.5 | **0.017** | 4.9 | **0.0106** |

|  |  | D | | P | | T | | O | | M | | Q | |
| --- | --- | --- | --- | --- | --- | --- | --- | --- | --- | --- | --- | --- | --- |
| Basin | Station | t statistic | Adj *p*-value | t statistic | Adj *p*-value | t statistic | Adj *p*-value | t statistic | Adj *p*-value | t statistic | Adj *p*-value | t statistic | Adj *p*-value |
| BS | N10 | 1.1 | 1 | -3.5 | 0.13 | 0.48 | 1 | 2.9 | 0.16 | 1.06 | 1 | 0.23 | 1 |
| BS | N11 | -0.93 | 1 | -6.1 | **0.0059** | -3.0 | 0.38 | 2.8 | 0.36 | -4.6 | **0.0081** | -2.9 | 0.35 |
| BS | N16 | 0.58 | 1 | -3.9 | 0.102 | 1.6 | 1 | 7.7 | **0.0015** | 0.9007 | 1 | -0.32 | 1 |
| BS | N17 | 2.6 | 0.58 | -2.5 | 0.4 | -0.603 | 1 | 7.2 | **0.00026** | -3.4 | 0.098 | -4.06 | 0.096 |
| BS | N18 | 2.06 | 0.902 | -1.9 | 1 | 0.039 | 1 | 6.7 | **0.00087** | -1.7 | 0.96 | -0.81 | 1 |
| BS | N21 | 5.2 | **0.024** | -3.3 | 0.18 | -1.8 | 1 | 3.9 | 0.087 | -5.02 | **0.017** | -3.9 | 0.109 |
| BS | N24 | 2.6 | 0.44 | -3.6 | 0.099 | 1.4 | 1 | 10.3 | **0.00031** | -2.3 | 0.43 | -1.4 | 1 |
| BS | N25 | 1.5 | 1 | -3.3 | 0.12 | 2.4 | 0.61 | 6.2 | **0.0012** | -0.52 | 1 | -0.31 | 1 |
| BP | Norrtalje | 0.22 | 1 | 0.0095 | 1 | 0.53 | 1 | -0.4 | 1 | 0.69 | 1 | -0.15 | 1 |
| BP | 6010 | -0.72 | 1 | -2.7 | 0.22 | -2.5 | 0.48 | 2.4 | 0.43 | -1.4 | 1 | 0.5 | 1 |
| BP | 6004 | -2 | 0.902 | -0.19 | 1 | -2.4 | 0.6005 | 1.3 | 1 | -6.001 | **0.000025** | -1 | 1 |
| BP | 6023 | -0.47 | 1 | 0.57 | 1 | -0.15 | 1 | -0.034 | 1 | 0.108 | 1 | -1.9 | 1 |
| BP | 6016 | 1.5 | 1 | -0.055 | 1 | 0.74 | 1 | -1.02 | 1 | -0.18 | 1 | -1.2 | 1 |
| BP | 6006 | -0.47 | 1 | -0.46 | 1 | 0.0049 | 1 | 2 | 0.77 | -2.6 | 0.205 | -0.96 | 1 |
| BB | BY2 | -1.6 | 1 | -0.27 | 1 | 0.25 | 1 | -0.12 | 1 | 1.9 | 0.87 | 0.75 | 1 |
| SK | SK3 | 2.03 | 0.92 | 0.11 | 1 | 2.2 | 0.94 | 1.8 | 1 | 7.9 | **0.0032** | 0.18 | 1 |
| SK | SK26 | 3.05 | 0.24 | 3.2 | 0.22 | -0.8 | 1 | 2 | 0.79 | 2.7 | 0.23 | 1.7 | 1 |
| SK | LYSE8 | 2.4 | 0.69 | 0.34 | 1 | 3.3 | 0.28 | 1.6 | 1 | 3.7 | 0.098 | 1.05 | 1 |
| SK | SK2 | 5.8 | **0.0026** | 1.5 | 1 | 3.5 | 0.17 | 1 | 1 | 4.5 | 0.088 | 4.5 | **0.042** |
| SK | SK24 | 3.8 | 0.069 | 1.4 | 1 | 1.2 | 1 | 2.4 | 0.46 | 6.3 | **0.003** | 1.3 | 1 |
| SK | SK21 | 2.6 | 0.44 | 2.2 | 0.73 | 1.2 | 1 | 1.8 | 1 | 5.4 | **0.019** | 1.09 | 1 |
| SK | SK1 | 1.4 | 1 | 1.6 | 1 | 1.1 | 1 | -0.34 | 1 | 3.6 | 0.13 | 4.9 | 0.0501 |

Table S4. SIMPER analysis results of significantly changed abundance between 1980’s and 2010’s and basin where the change occurred. Significance: * *p*<0.05, ** *p*<0.01, *** *p*<0.001. Basin BS Bothnian Sea, BP Baltic Proper, BB Bornholm Basin, SK Skagerrak.

| **Species** | **Basin** |
| --- | --- |
| Abra alba ** | SK |
| Abra nitida ** | SK |
| Acaulis primarius ** | SK |
| Amaeana trilobata *** | SK |
| Amblyops abbreviatus ** | SK |
| Ampelisca macrocephala *** | SK |
| Ampelisca spinipes ** | SK |
| Ampharete baltica *** | SK |
| Amphicteis gunneri *** | SK |
| Amphilepis norvegica * | SK |
| Amphilochoides serratipes *** | SK |
| Amphiura *** | SK |
| Amythasides macroglossus ** | SK |
| Anobothrus gracilis *** | SK |
| Aoridae *** | SK |
| Aphelochaeta mcintoshi ** | SK |
| Apherusa bispinosa ** | SK |
| Aphrodita aculeata ** | SK |
| Apistobranchus tenuis ** | SK |
| Apseudes spinosus *** | SK |
| Araphura filiformis ** | SK |
| Arctica islandica *** | SK |
| Argissa hamatipes * | SK |
| Aricidea cerrutii * | SK |
| Artacama *** | SK |
| Astarte elliptica * | SK |
| Asterias rubens ** | SK |
| Asterope mariae *** | SK |
| Axinulus eumyarius ** | SK |
| Baeonectes muticus ** | SK |
| Bathyarca pectunculoides *** | SK |
| Bathymedon saussurei ** | SK |
| Bathyporeia pilosa ** | BP |
| Bela nebula ** | SK |
| Brachydiastylis resima ** | SK |
| Brachyura ** | SK |
| Brada villosa *** | SK |
| Brissopsis lyrifera * | SK |
| Buccinum undatum *** | SK |
| Byblis gaimardii *** | SK |
| Bylgides sarsi *** | BS/BP |
| Callinera buergeri ** | SK |
| Calocarides coronatus *** | SK |
| Calocaris macandreae ** | SK |
| Campylaspis costata *** | SK |
| Capitella capitata ** | SK |
| Caudofoveata *** | SK |
| Cerianthus lloydii * | SK |
| Chaetoderma nitidulum *** | SK |
| Chaetopterus variopedatus ** | SK |
| Chaetozone setosa *** | SK |
| Cheirocratus sundevallii *** | SK |
| Chone ** | SK |
| Chrysallida ** | SK |
| Cirratulidae *** | SK |
| Clausinella fasciata *** | SK |
| Clymenura borealis ** | SK |
| Conchoecia borealis ** | SK |
| Conchoecia elegans ** | SK |
| Conchoecia obtusata ** | SK |
| Cossura longocirrata ** | SK |
| Crangon ** | SK |
| Cucumariidae *** | SK |
| Cuspidaria obesa ** | SK |
| Cyanophthalma obscura * | BS/BB |
| Cylichna alba ** | SK |
| Cylichna cylindracea *** | SK |
| Dentalium ** | SK |
| Diaphana * | SK |
| Diastylis bradyi *** | SK |
| Diastylis lucifera *** | SK |
| Diastyloides serratus * | SK |
| Diplocirrus glaucus ** | SK |
| Dipolydora coeca * | SK |
| Drilonereis filum *** | SK |
| Dulichia ** | SK |
| Dyopedos porrectus ** | SK |
| Ebalia tuberosa ** | SK |
| Echinocardium ** | SK |
| Echinocardium cordatum *** | SK |
| Echinocythereis echinata ** | SK |
| Echiurus echiurus ** | SK |
| Edwardsiidae ** | SK |
| Einhornia crustulenta ** | SK |
| Entalina tetragona * | SK |
| Enteropneusta *** | SK |
| Ericthonius difformis *** | SK |
| Eriopisa elongata *** | SK |
| Eteone longa *** | SK |
| Euclymeninae *** | SK |
| Eudorella truncatula * | SK |
| Eugerda tenuimana ** | SK |
| Eulimidae ** | SK |
| Eumida bahusiensis * | SK |
| Eurycope cornuta ** | SK |
| Eurycope producta *** | SK |
| Euspira montagui *** | SK |
| Euspira nitida ** | SK |
| Exogone hebes *** | SK |
| Exogone verugera ** | SK |
| Fabriciola baltica ** | SK |
| Gattyana amondseni ** | SK |
| Genaxinus eumyarius *** | SK |
| Glycera alba *** | SK |
| Glycera rouxii *** | SK |
| Glycera unicornis *** | SK |
| Glycinde nordmanni *** | SK |
| Glyphohesione klatti *** | SK |
| Golfingia *** | SK |
| Goniada maculata *** | SK |
| Gyptis *** | SK |
| Haploops tubicola *** | SK |
| Harmothoe *** | SK |
| Harpinia crenulata ** | SK |
| Harrimania kupfferi ** | SK |
| Heteroclymene robusta ** | SK |
| Heteromastus ** | SK |
| Heteromastus filiformis ** | SK |
| Hilbigneris gracilis *** | SK |
| Hyperiidea *** | SK |
| Ischyrocerus ** | SK |
| Kelliella miliaris ** | SK |
| Labidoplax buskii *** | SK |
| Laetmonice filicornis *** | SK |
| Laonice cirrata *** | SK |
| Laonome kroyeri *** | SK |
| Leptostylis ** | SK |
| Leucon acutirostris ** | SK |
| Leucon nasica * | SK |
| Leucothoe lilljeborgii *** | SK |
| Levinsenia gracilis *** | SK |
| Liocarcinus depurator ** | SK |
| Lucinoma borealis *** | SK |
| Lysianassidae ** | SK |
| Lysilla loveni * | SK |
| Maera loveni * | SK |
| Maldane sarsi * | SK |
| Maldanidae ** | SK |
| Marenzelleria *** | BS/BP/BB |
| Megamphopus cornutus ** | SK |
| Melphidippa borealis ** | SK |
| Mendicula ferruginosa ** | SK |
| Monoporeia affinis *** | BS/BP |
| Monopseudocuma gilsoni ** | SK |
| Montacuta substriata ** | SK |
| Munnopsis typica ** | SK |
| Musculus niger *** | SK |
| Mya truncata *** | SK |
| Myriochele ** | SK |
| Mysida *** | BS |
| Natica ** | SK |
| Nemertea *** | BP/BB |
| Neohela monstrosa *** | SK |
| Nephtys ** | SK |
| Nephtys ciliata *** | SK |
| Nephtys hombergii *** | SK |
| Nothria conchylega ** | SK |
| Nucula tumidula *** | SK |
| Nudibranchia *** | SK |
| Oligochaeta * | BP/BB |
| Onchnesoma steenstrupi ** | SK |
| Onoba ** | SK |
| Ophelina acuminata *** | SK |
| Ophelina modesta *** | SK |
| Ophiocten affinis *** | SK |
| Ophiura *** | SK |
| Ophiura albida *** | SK |
| Ostracoda *** | SK |
| Owenia fusiformis ** | SK |
| Oxydromus flexuosus ** | SK |
| Pagurus bernhardus ** | SK |
| Paradiopatra quadricuspis *** | SK |
| Paradoneis armata *** | SK |
| Paradoneis eliasoni ** | SK |
| Paradulichia typica ** | SK |
| Parexogone hebes ** | SK |
| Pariambus typicus ** | SK |
| Pectinaria ** | SK |
| Pedicellaster typicus *** | SK |
| Pennatula phosphorea * | SK |
| Perioculodes longimanus ** | SK |
| Phaxas pellucidus *** | SK |
| Pherusa plumosa ** | SK |
| Philine *** | SK |
| Philomedes brenda * | SK |
| Pholoe minuta *** | SK |
| Phoronis muelleri * | SK |
| Photis longicaudata ** | SK |
| Photis reinhardi ** | SK |
| Phtisica marina ** | SK |
| Phyllodoce mucosa *** | SK |
| Phyllodoce rosea *** | SK |
| Phylo kupfferi ** | SK |
| Phylo norvegica *** | SK |
| Piscicola geometra ** | SK |
| Pista cristata *** | SK |
| Poecilochaetus serpens ** | SK |
| Polydora ciliata *** | SK |
| Polyphysia crassa *** | SK |
| Priapulus caudatus *** | SK |
| Prionospio cirrifera *** | SK |
| Prionospio fallax *** | SK |
| Prionospio steenstrupi ** | SK |
| Protomystides exigua ** | SK |
| Pseudamussium peslutrae ** | SK |
| Pseudocuma simile ** | SK |
| Radix balthica * | SK |
| Retusa umbilicata ** | SK |
| Rhaphidrilus ** | SK |
| Rhodine gracilior *** | SK |
| Rhodine loveni * | SK |
| Saduria entomon ** | BB |
| Scina borealis ** | SK |
| Scolelepis tridentata *** | SK |
| Scoletoma fragilis *** | SK |
| Scoletoma impatiens *** | SK |
| Scoloplos armiger ** | SK |
| Sipuncula ** | SK |
| Skogsbergia megalops ** | SK |
| Sosane sulcata ** | SK |
| Spatangidae *** | SK |
| Sphaerodoropsis philippi ** | SK |
| Sphaerodorum flavum *** | SK |
| Spiochaetopterus * | SK |
| Spiophanes bombyx *** | SK |
| Spisula subtruncata ** | SK |
| Sthenelais limicola *** | SK |
| Streblosoma bairdi ** | SK |
| Syllidae ** | SK |
| Syllis cornuta ** | SK |
| Syllis variegata ** | SK |
| Tanaidacea ** | SK |
| Taranis moerchi *** | SK |
| Tellimya ferruginosa *** | SK |
| Tellimya tenella ** | SK |
| Tharyx killariensis *** | SK |
| Themisto abyssorum *** | SK |
| Theodoxus fluviatilis * | BP |
| Thracia convexa *** | SK |
| Thyasira ** | SK |
| Thyasira obsoleta *** | SK |
| Thyasira sarsii *** | SK |
| Tritia nitida ** | SK |
| Tryphosites longipes ** | SK |
| Turbellaria *** | SK |
| Virgularia mirabilis *** | SK |
| Xenodice frauenfeldti ** | SK |
| Yoldiella lucida ** | SK |
| Yoldiella nana ** | SK |
